# Supplementary material for: Prior knowledge promotes hippocampal separation but cortical assimilation in the left inferior frontal gyrus
Source: Nat Commun. 2020 Sep 14;11:4590. doi: 10.1038/s41467-020-18364-1 (PMC7490707; doi:10.1038/s41467-020-18364-1)
Supplement: Supplementary file 1 — Supplementary Information [file 41467_2020_18364_MOESM1_ESM.pdf]

Supplementary Information

Prior knowledge promotes hippocampal separation but cortical assimilation in the left inferior frontal gyrus

Bein, O., Reggev, N. and Maril, A.

This supplemental includes:

Supplementary Figures 1-6.

Supplementary Tables 1-2.

Supplementary Notes 1-11.

Supplementary Figures

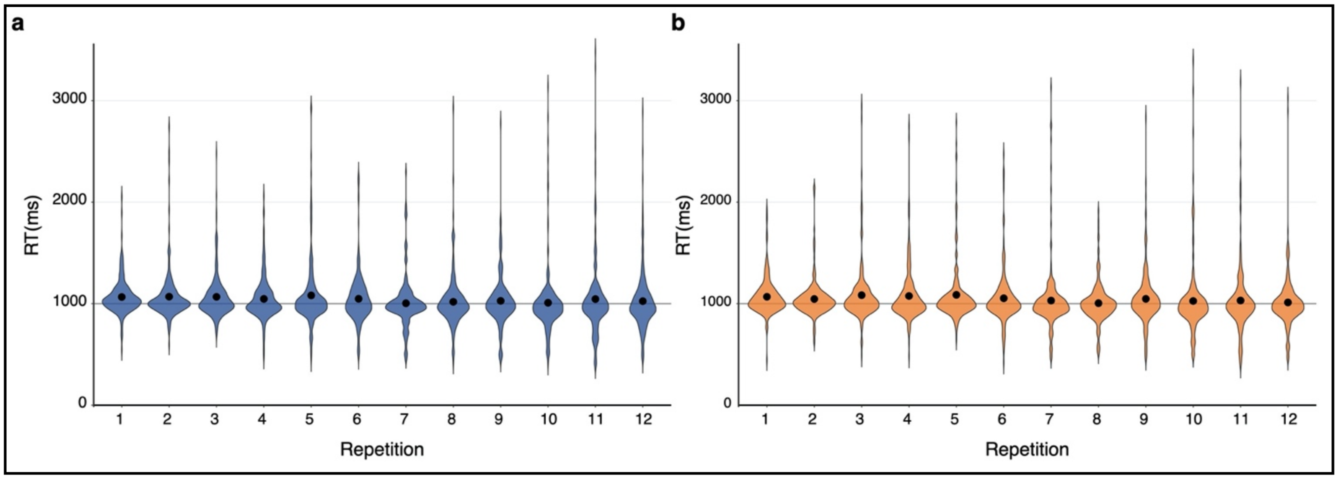

Supplementary Figure 1. Reaction times (RT) during the associative learning task in each repetition cycle. a. RT for the prior knowledge pair type (PK; blue). b. RT for the no prior knowledge pair type (n-PK; orange). The black dots indicate mean RT. See related Supplementary Note 1 for further details and statistical analysis. N=19. Source data are provided as a Source Data file.

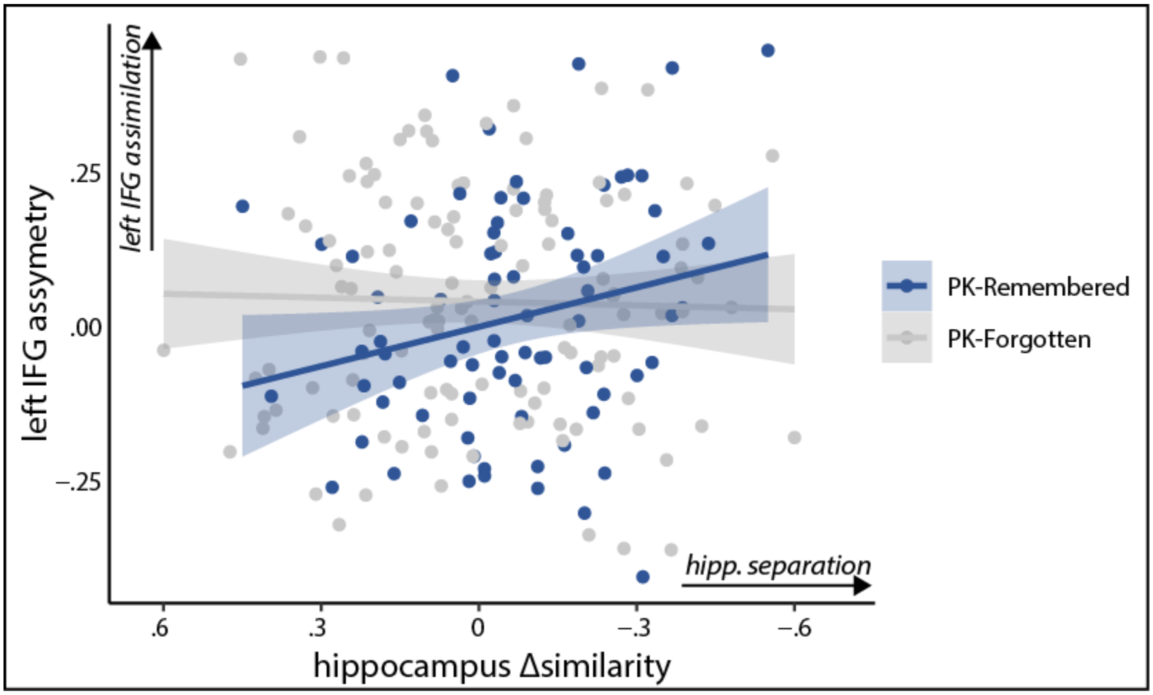

Supplementary Figure 2. Hippocampal (hipp.) separation correlated with left inferior frontal gyrus (left IFG) assimilation. PK: prior knowledge pairs, including a famous face and a novel face. ΔSimilarity: similarity post-learning minus similarity pre-learning. Asymmetry: asymmetric changes in similarity. Data are from PK pairs only, Remembered: pairs subsequently correctly remembered with high-confidence in the subsequent memory test. Forgotten: pairs that were incorrectly identified in the memory test. See related Supplementary Note 2 for details and statistical analysis. N=18, dots reflect ΔSimilarity/asymmetry between pairs of faces. The lines reflect a linear regression, ribbons reflect 95% CI.

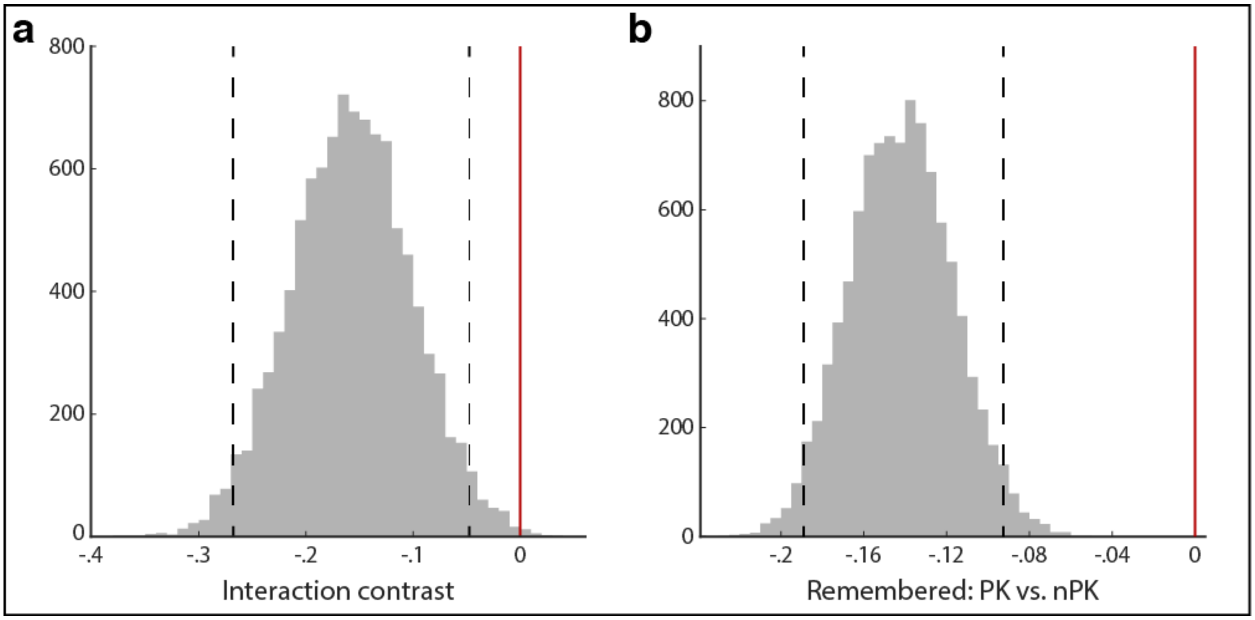

Supplementary Figure 3. Distribution of similarity in the left anterior hippocampus (subsampled trials, total of 10,000 iterations). a. Interaction contrast values (PK/nPK by remembered/forgotten). b. PK vs. nPK differences for remembered trials. Dotted lines reflect the 95% confidence interval, the 0 line is marked in red. See related Supplementary Note 4 for more details.

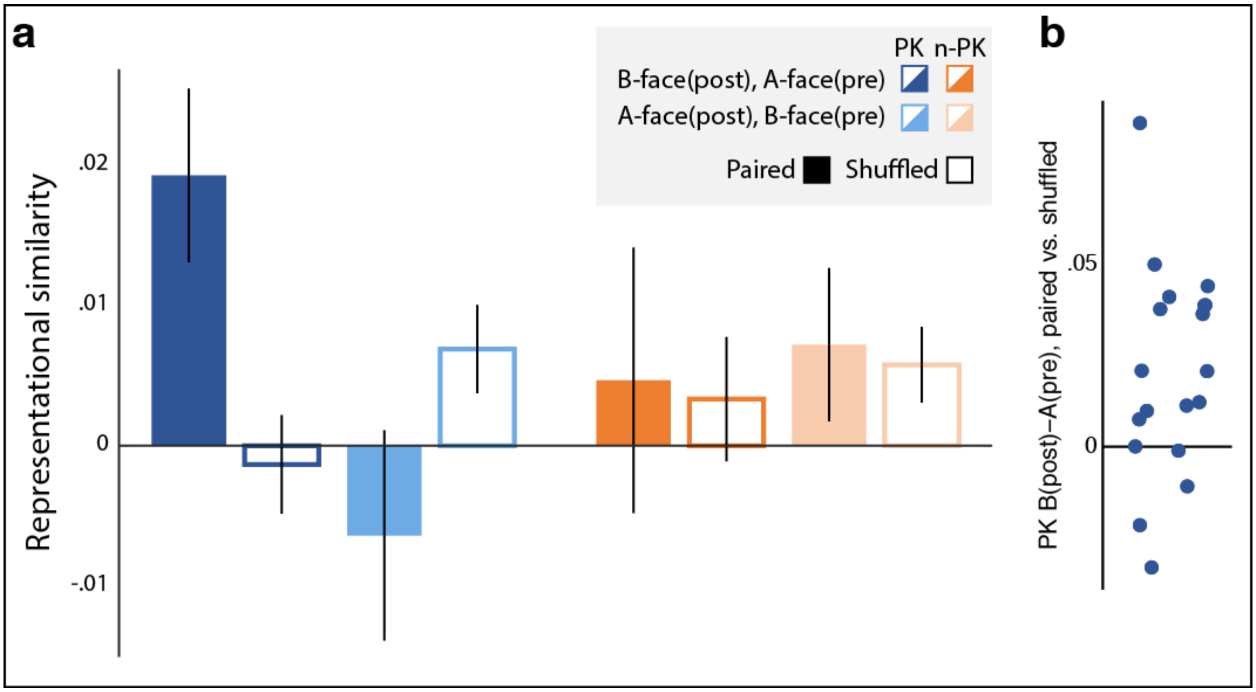

Supplementary Figure 4. Similarity values comprising the asymmetry measure, left inferior frontal gyrus. a. similarity values. “pre”: pre-learning, “post”: post-learning. PK: prior knowledge pairs. N-PK: no prior knowledge pairs. Paired: similarity between faces that were paired during the associative learning task. Shuffled: similarity between the same faces, but the faces are shuffled so that the similarity is computed between faces that did not appear together during the associative learning task. N=19. Data are presented as mean values, error bars reflect +/- SEM. b. dot plots reflect individual participants’ differences between the similarity of B-face(post) and A-face(pre) for paired and shuffled faces, in the PK pair type (i.e., the difference between the first and second bars from the left in a). Source data are provided as a Source Data file. See Supplementary Note 6 for details and statistical analysis.

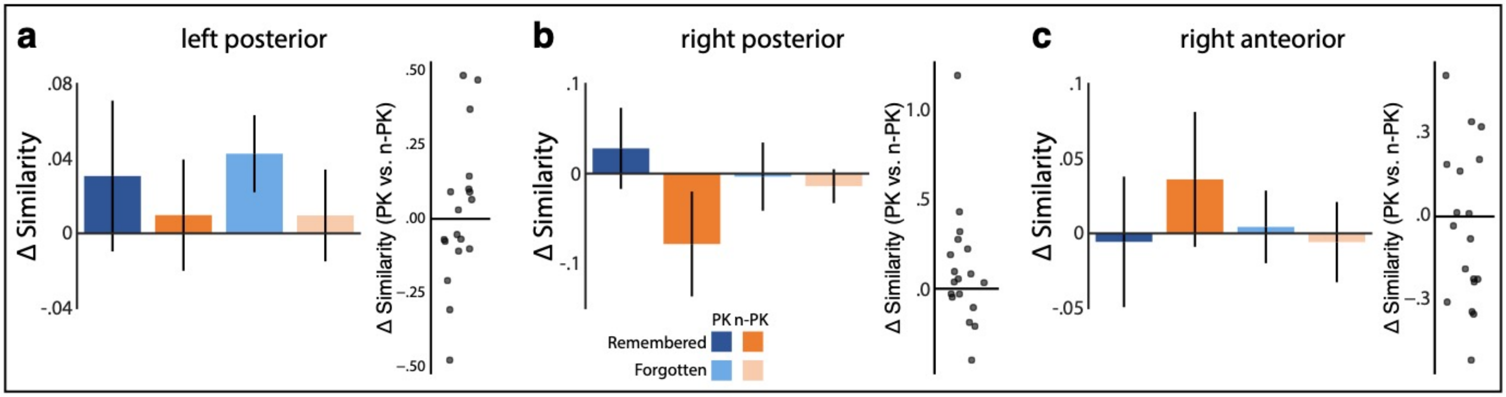

Supplementary Figure 5. Representational similarity changes in hippocampus ROIs. a. left posterior hippocampus. b. right posterior hippocampus. c. right anterior hippocampus. The results from the left anterior hippocampus are reported in the main text. ΔSimilarity: similarity post-learning minus similarity pre-learning. PK: prior knowledge pairs, n-PK: no prior knowledge pairs. Dot plots reflect individual participants' Δ Similarity reduction due to Prior Knowledge (PK remembered pairs minus n-PK remembered pairs), as was displayed in Figure 2 in the main text. N=18. Data in the bar graphs are presented as mean values, error bars reflect +/- SEM. Source data are provided as a Source Data file. See Supplementary Note 8 for details and statistical analysis.

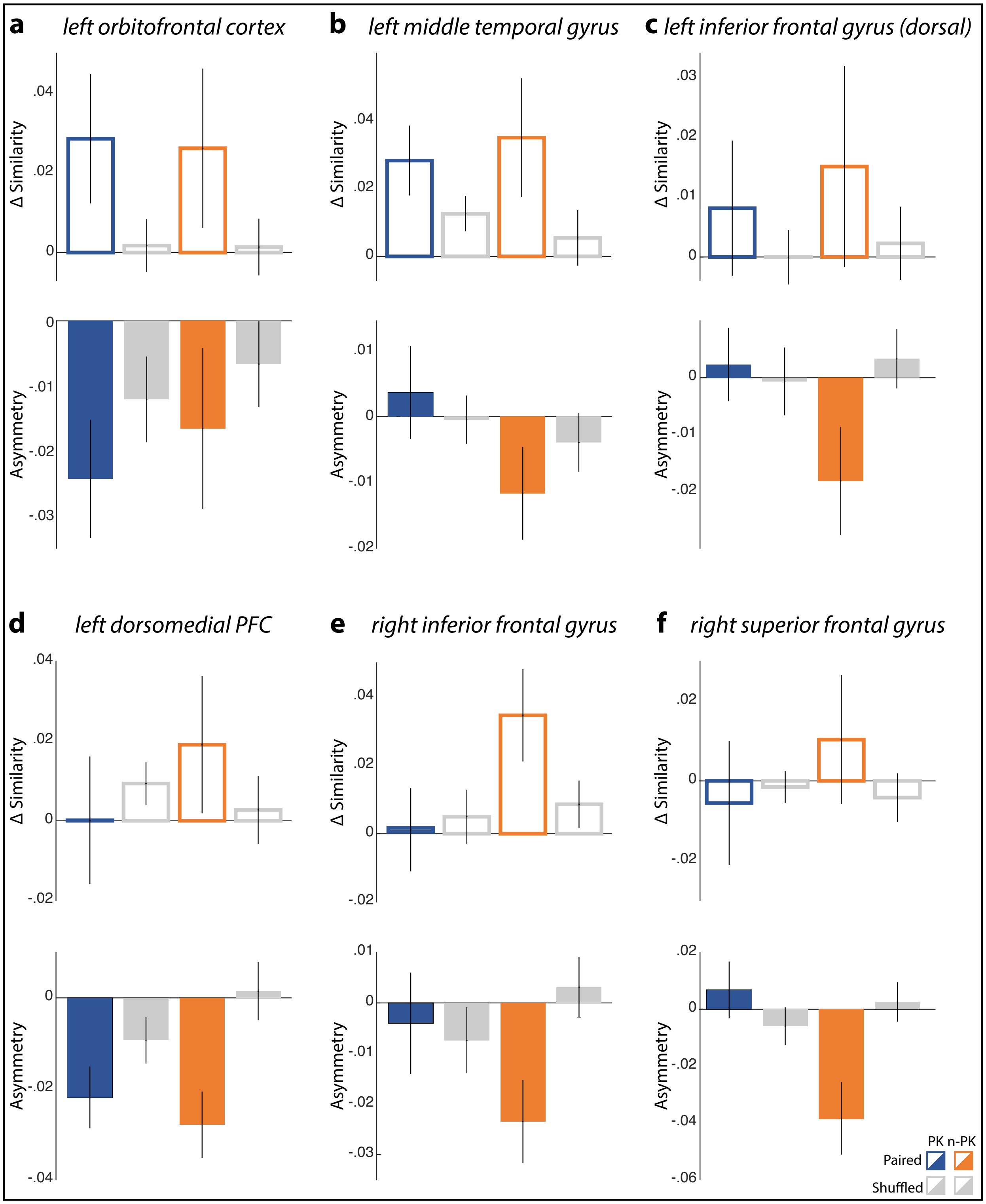

Supplementary Figure 6. Similarity results in cortical ROIs demonstrating functional connectivity with the left anterior hippocampus. Δ Similarity: the similarity difference from before to after learning. Asymmetry: asymmetric changes in similarity. a. left orbital frontal cortex. b. left middle temporal gyrus. c. left inferior frontal gyrus (dorsal cluster). d. left dorsomedial prefrontal cortex (PFC). e. right inferior frontal gyrus. f. right superior frontal gyrus. iN=19. Data are presented as mean values, error bars reflect +/- SEM. Source data are provided as a Source Data file. See Supplementary Note 9 for details and statistical analysis.

Region	*MNI coordinates* *x*	*y*	*z*	*Z value*	*Num. Voxels*
Dorsomedial prefrontal cortex	6	36	50	3.92	1042
L orbitofrontal cortex	-24	48	-14	3.67	170
L middle temporal gyrus	-44	0	-30	3.66	568
R superior frontal gyrus	10	62	28	3.50	62
R inferior frontal gyrus	42	32	-22	3.38	269
L inferior frontal gyrus (ventral)	-48	30	-16	3.27	87
L inferior frontal gyrus (dorsal)	-52	30	10	3.00	70
L angular gyrus	-42	-48	26	3.19	126
Cerebellum	-24	-88	-42	2.94	67

Supplementary Table 1. Regions demonstrating greater functional connectivity (gPPI) with the left anterior hippocampus for prior knowledge (PK) pairs compared to no prior knowledge (n-PK) pairs during associative learning. No region was observed in the opposite contrast (see main text, Methods, Results, and Figure 3).

Region	*MNI coordinates* *x*	*y*	*z*	*Z value*	*Num. Voxels*
PK:1st > n-PK:1st					
vmPFC	-10	48	-22	4.20	60
n-PK:1st > PK:1st					
left precuneus	-12	-40	44	3.78	85
n-PK: all > PK: all					
middle temporal gyrus	44	-32	-4	4.10	97
left middle frontal gyrus	-30	40	12	3.87	190
right middle frontal gyrus	22	58	28	3.65	153
left lingual gyrus	-6	-70	-2	3.64	65

Supplementary Table 2. Univariate activation during the associative learning task. PK: prior konwledge pairs, including famous and novel faces. N-PK: no prior knowledge pairs, including two novel faces. 1st: only first presentation of the pairs. All: all repetition of the pairs.

Supplementary Notes

Supplementary Note 1 (related to Supplementary Fig. 1): behavioral results from the associative learning task. During the associative learning task, participants viewed pairs of faces. Some pairs contained a famous and a novel face (prior knowledge, PK) and some pairs included two novel faces (no prior knowledge, n-PK). The pairs were repeated 12 times in cycles, and each cycle included all pairs in random order. For each pair, participants judged whether both faces were of the same gender (experimental trials were always of the same gender; we interspersed mixed-gender pairs as fillers to make the task possible, see Methods). Participants performed at ceiling for the gender judgements across all pair types and repetition cycles (PK and n-PK, same- and mixed-gender pairs; all > 96%). The mixed-gender pairs were not further analyzed. We further excluded from all analyses PK pairs in which participants did not recognize the famous face in the post-experiment knowledge questionnaire (for six participants, one pair was removed; the pairs included different famous faces across these participants). For analysis of the reaction times (RTs) during learning, we first excluded all incorrect responses and trials in which participants responded more than once. We further excluded, for each participant, trials in which RTs deviated more than 3 standard deviations from the mean in each cycle of repetition. On average, 8.42 and 5.53 responses (6% and 4%) were excluded for PK and n-PK pairs, respectively. Participants demonstrated learning, as indicated by faster RTs towards the end of learning than early in learning (Supplementary Figure 1).

For statistical analysis, single-trial RTs were entered as the predicted variable to general linear mixed models (gLMM, as implemented by the glmer function, lme4 package in R[1]) with inverse Gaussian distribution as the linking distribution function[2]. Model comparisons were used to examine the effects of Pair Type (PK/n-PK), Repetition (1-12 as a continuous variable), and their interaction. All models included a random intercept per participant. We found that Repetition significantly predicted the decrease in RT, indicating learning. Specifically, a model including the Repetition and Pair Type factors significantly outperformed a model that included only Pair Type, indicating that Repetition significantly explained variance in RTs ($\chi^2 = 105.58$, $p < .0001$, AIC difference: 104, BIC difference: 97). Repetition significantly explained RTs when a simpler model was conducted, which included Repetition as a single fixed-effect, and when this

model was compared to a null-model that only included a random intercept per participant and no fixed effect (χ^2 = 105.3, p < .0001, AIC difference: 103, BIC difference: 97). There was also a small effect of Pair Type, suggesting that participants responded to PK pairs slightly faster than to n-PK pairs. This effect was significant when comparing the full model, including Pair Type and Repetition, versus a model including only Repetition (χ^2 = 3.86 , p = .049, with a minor AIC difference of 2, but BIC difference of -5), and marginally significant when comparing the simpler model, including only Pair Type, to the null model (χ^2 = 3.58 , p = .058, with a minor AIC difference of 1, but BIC difference of -5). There was no Pair Type by Repetition interaction, as indicated by comparing a model with Pair Type, Repetition, and their interaction, to the same model without the interaction term (χ^2 = .18, p = .67).

During the pre- and post-learning scans, participants made male/female judgments for single faces that appeared in the associative learning task. We term faces that appeared as the first face in a pair during the learning task A-faces and faces that appeared second B-faces. Accuracy was at ceiling during the pre-scan and the post-scan. On average, participants responded to all faces with 96-99% accuracy during both pre-learning and post-learning scans (rates for all types of faces, e.g., famous faces and novel faces did not differ between pre- and post-learning scans.)

Supplementary Note 2 (related to Supplementary Figure 2): Asymmetry in the left IFG might be correlated with hippocampal separation. In the main text we report asymmetrical changes in representation similarity for PK pairs in the left inferior frontal gyrus (left IFG; see main text for more details). In PK pairs, but not in n-PK pairs, the novel B-face became more similar to the famous A-face through learning than vice-versa. This result suggests assimilation into prior knowledge structures. In the hippocampus, we report that PK pairs that participants later remembered in an associative memory test, but not those that participants forgot, became more separated (less similar) through learning. Here, we examine whether assimilation in the left IFG might be correlated with hippocampal separation. We used a linear mixed-level model approach (lmer function, lme4 package in R[1]), where trial-by-trial asymmetry scores in the left IFG were the explained variable, and similarity differences from pre-learning to post-learning in

the hippocampus were the explaining variable. We only looked at PK pairs, in which assimilation occurred, and we broke this analysis to remembered (high-confidence responses only, as in the main analysis) and forgotten pairs, as hippocampal separation was only observed for remembered pairs. Thus, if any relationship exists between hippocampal separation and left IFG assimilation, we should expect to find it in remembered pairs. Statistical significance was determined by comparing a model including the factor of interest to an identical model excluding the factor of interest. All models included an intercept per participant. First, we examined only remembered pairs and found that similarity changes in the hippocampus significantly explained variance in asymmetry in the left IFG (χ^2 = 4.15, p = .04, AIC reduction: 2, equivalent BIC scores: difference of +.19, see Supplementary Figure 2). We additionally ran two control analyses. First, we examined specificity to remembered trials, by including both remembered and forgotten pairs and testing the interaction of memory (remembered vs. forgotten) with hippocampal similarity. This interaction was marginally significant (χ^2 = 3.41, p = .06, AIC reduction: 1.5, but BIC increase: 1.9). Then, we also examined whether the observed correlation was specific to paired faces, namely, faces that appeared together during the associative learning task. For both for the left IFG and the hippocampus, we took for each pair, the asymmetry (or similarity difference in the hippocampus) computed between the A-face and its paired B-face, and subtracted from it the same measure, but computed between that A-face and all other B-faces that appeared in with other PK A-faces (i.e., we subtracted the asymmetry/similarity differences for shuffled pairs). Taking this measure that is a pair-specific measure, we again obtained a marginal correlation (χ^2 = 3.37, p = .06, AIC reduction: 1.5, but BIC increase: 1). Together, these results provide some preliminary evidence that indeed pairs with higher hippocampal separation, also exhibited larger asymmetry in the left IFG. However, we note that the correlation is moderate, and the control analyses did not reach significance. Another study, potentially with more statistical power, might better elucidate this relationship.

Supplementary Note 3. Control for univariate activation. To rule out the possibility that univariate activation accounted for our similarity findings, we included univariate activation during the pre and post similarity scans as a factor in multiple regression models. For each

participant in each of the relevant ROIs, the faces' t-maps were binned and averaged in each pair type (PK/nPK) and by A/B-faces. For the memory analysis, maps were further divided to remembered pairs (high-confidence hits) and forgotten pairs (misses), paralleling the similarity analysis.

In the left anterior hippocampus, we controlled for univariate activation in the Prior Knowledge by Memory interaction by including univariate activation in models implemented via ANOVA (aov function in R, stats package). Similarity differences per participant in each of the four bins (PK/n-PK by remembered/forgotten) were the explained variables. As explaining variables of interest, we included Prior Knowledge (PK/n-PK), Memory (Remembered/Forgotten), and their interaction. Since our similarity measures are a difference between post and pre-similarity, in the first model we controlled for the difference in univariate activation between post and pre similarity, by including in the model these differences in each of the four bins (PK/n-PK by remembered/forgotten), for both A-faces and B-faces. The models further included a within-participant error term for each of the factors of interest (i.e., participant/Prior Knowledge*Memory). The Prior Knowledge by Memory interaction was significant ($F_{(1,15)}$= 9.44, p = .008, η_p^2 = .39). In a second model, instead of activation differences, we included activation for pre and post scans separately (four variables: A/B face by pre-/post-learning). Again, a significant interaction was obtained ($F_{(1,13)}$= 10.05, p = .007, η_p^2 = .43). We proceeded to the simple effect obtained between remembered PK pairs and remembered n-PK pairs. We repeated the same models as before, controlling for the difference in univariate activity between pre- and post-learning in one model, and each phase separately in another model, but including only values of remembered pairs (for similarity and univariate data). In both models, an effect of Prior Knowledge was revealed (control for pre-post differences: $F_{(1,15)}$= 9.90, p = .007, η_p^2 = .40; each phase: $F_{(1,13)}$ = 9.10, p = .01, η_p^2 = .41). Then, to test for the simple effects of Memory within each pair type, we repeated the same two models as before, but now taking first only PK pairs, then only n-PK pairs (remembered vs. forgotten pairs, now only a Memory variable was included in addition to the univariate variables). The effect of Memory was significant in all four models (significant in PK pairs, pre-post differences: $F_{(1,15)}$ =

6.04, p = .03, η_p^2 = .29; each phase: $F_{(1,13)}$ = 5.98, p = .03, η_p^2 = .32; marginally significant in n-PK pairs: $F_{(1,15)}$ = 3.38, p < .09, η_p^2 = .18; each phase: $F_{(1,13)}$ = 3.64, p < .08, η_p^2 = .22).

In the left inferior frontal gyrus, the asymmetry measure in PK pairs was different from 0, as well as from the asymmetry in shuffled pairs. Thus, in the current analyses, we wished to control for univariate activation for these two comparisons. As before, we accounted for univariate activation of A- and B-faces. Note, however, that both paired and shuffled pairs were computed using the same A and B faces, but with these faces paired differently to compute the asymmetry. Therefore, there was no separate univariate activation for A- and B-faces in paired and shuffled pairs, as both included the same faces. Thus, to allow us to examine whether the difference in asymmetry between paired and shuffled faces would holds when controlling for univariate activation of the A and B faces, we needed one asymmetry measure per participant. To that end, for each participant, we subtracted the asymmetry measure in the shuffled faces baseline from the asymmetry measure for paired faces. This difference measure was then taken as the explained variable when comparing the difference in asymmetry for paired versus shuffled pairs. In additional models, we also examined the raw asymmetry measure (to control for univariate activity for the comparison against 0). As the explaining variables, we included as before either the pre-post differences for A and B faces, or each of the pre and post phases separately. This yielded at total of four models (difference from 0/difference from shuffled pairs by activation difference/each phase separately). The linear regression models were evaluated using the lm function in R (stats package; note that the explained variable is already a within-participant difference measure, hence there is no need to include a within-participant error term, as was done above). In all four models, the asymmetry measure was significant (the group intercept, paired-shuffled, pre-post differences: β = 0.029, $t_{(16)}$ =3.66, p = .002; each phase: β = 0.037, $t_{(14)}$ = 4.46, p < .001; paired-0, pre-post differences: β = 0.026, $t_{(16)}$ =2.65, p = .017; each phase: β = 0.029, $t_{(14)}$ = 3.23, p = .006).

Taken together, these analyses confirm that our similarity findings are unlikely to be attributed to differences in univariate activation.

Supplementary Note 4 (related to Supplementary Figure 3). Control for number of trials in the hippocampal results. In the left anterior hippocampus, we found an interaction of PK by Memory. Specifically, PK pairs that were later remembered became more separated with learning, while n-PK pairs that were later remembered became more similar. These similarity changes we specific to remembered pairs, as pairs that were later forgotten did not demonstrate any changes in similarity (See Results, main text). Here, we examined whether differences in the number of trials that participants remembered (and therefore forgot) could account for our results.

Specifically, for each participant, we identified the condition with the least number of trials across the 4 conditions (PK/n-PK by remembered/forgotten), and randomly selected the same number of trials in the other conditions. Then, we computed the average pre-post similarity difference across the subsampled trials in each condition (as was done in the main analysis). This step was repeated 10,000 iterations to construct a distribution.

Our main interest was in the interaction of Prior Knowledge (PK/n-PK) by Memory (remembered/forgotten). To that end, we computed the interaction contrast of the interaction ([PK:remembered-PK:forgotten]-[n-PK:remembered-n-PK:forgotten]), per participant and then averaged at the group level, per iteration. We found that even with subsampling trials, 99.8% of the contrasts were lower than zero (Supplementary Figure 3; Note that since in the PK:remembered condition we found separation, i.e., a negative similarity values, we would expect the contrast to be negative). We have further tested the comparison between PK:remembered and
n-PK:remembered, by computing the distribution of group average difference between these conditions (specifically, PK:remembered minus n-PK:remembered). We found that the group average differences in all 10,000 iterations were negative (Supplementary Figure 3: negative values were expected due to the separation in the PK:remembered condition, as in the interaction contrast).

In addition, we examined whether the specificity to remembered trials was obtained in this analysis as well, in either the PK pairs or the n-PK pairs. Within each pair type, we computed the group average difference between remembered and forgotten pairs, in each of

the 10,000 iterations. For PK pairs, 96.4% of the values were below zero, consistent with our main result that remembered pairs became less similar than forgotten. In the n-PK pair type, 98.6% of the values were above zero, consistent with our main result that for n-PK pairs, remembered pairs became more similar than forgotten.

Together, these results suggest that our findings are unlikely to be attributed to differences in the number of trials per condition.

Supplementary Note 5. Representational similarity before and after learning in the left anterior hippocampus. In the main text, we present the difference between similarity before learning and similarity after learning. Here, we present the similarity values from the pre-learning and the post-learning scans separately, for completeness. We note that as previous studies examining pre- to post-learning differences in similarity[3–5], the current study was not designed to look these similarity values separately. The similarity values before and after learning are influenced by several factors such as the sluggish nature of the BOLD signal, processing steps and correlations between the regressors in the fMRI GLM, that are dependent on the order of the stimuli as presented in the experiment[6,7]. Therefore, the raw similarity values are difficult to interpret at face value. Pattern similarity studies with various designs have different ways by which they address these issues, specific to each study and their aims. Previous studies that examined differences due to learning have typically addressed the aforementioned concerns by having the pre-learning and post-learning scans identical, subtracting the similarity values before learning from those values after learning, and reporting a difference in similarity[3–5]. This way, any influences from the BOLD signal, preprocessing, or correlations between GLM regressors are removed by the subtraction. The difference in similarity from before to after learning can therefore be interpreted with confidence. As detailed in the Methods, we followed the same approach in the current study. We further note that even though we did counterbalance the order of PK and n-PK faces across participants (see Methods), we still had no control over which pairs the participants later remembered or forgot.

Nevertheless, we acknowledge that it might be interesting to examine whether prior knowledge influence how neural patterns prior to associative learning might impact successful

encoding, as measured by subsequent memory for the associated pair. To facilitate future research, we report here similarity values before and after learning separately, acknowledging that any findings, if emerges, should be confirmed in another design. As a reminder, when looking at differences from before to after learning, we found that for remembered pairs, the neural representations of faces in PK pairs became less similar to each other through learning, while the representations of faces in the n-PK pairs became more similar (Results, main text). When looking at similarity values in the post-learning scans, the similarity between remembered PK pairs was lower than in the n-PK pairs (PK: M = .02, SD = .07; n-PK: M = .09, SD = .12). We additionally found that in the pre-learning scans, the similarity was higher between remembered PK pairs compared to remembered n-PK pairs (PK: M = .08, SD = .10; n-PK: M = .10, SD = .11). Repeated-measures ANOVA, with the factors of Prior Knowledge (PK/n-PK) and Time Point (pre-learning/post-learning) revealed a significant interaction of Prior Knowledge and Time Point ($F_{(1,17)} = 8.88$, $p = .008$; simple-effects: pre-learning, PK vs. n-PK: $t_{(1,17)} = 1.95$, $p = .07$; post-learning, PK vs. n-PK: $t_{(1,17)} = 1.90$, $p = .07$; PK: pre- vs. post-learning: $t_{(1,17)} = 3.01$, $p = .01$; n-PK: pre- vs. post-learning: $t_{(1,17)} = 2.08$, $p = .05$). The same ANOVA for forgotten pairs revealed no main effect nor an interaction (all $F_{(1,17)}$'s < 1.65, p's > .21). Hippocampal neural patterns prior to learning have been shown to modulate subsequent learning in rodents and humans [8–10]. It is an interesting possibility that prior knowledge might modify the preconditions that determine whether future associative learning will be successful or not. However, even though our experiment was counterbalanced, for the reasons discussed above, it is currently unclear whether the neural patterns prior to learning reflect a true difference in neural similarity. Future research, using a methodology that enables a careful examination of the before and after learning values separately, potentially in a slow event-related design or other imaging techniques, could better elucidate these preliminary findings. Importantly, our results show that similarity differences from before to after learning cannot be attributed to differences before learning alone, as after learning, the similarity between remembered PK pairs was lower than n-PK pairs.

Supplementary Note 6 (related to Supplementary Figure 4). Similarity values comprising the asymmetry measure in the left inferior frontal gyrus. In the main text we report asymmetry in representational changes in the left inferior frontal gyrus (left IFG). Specifically, we defined asymmetry as the difference between (1) the similarity of the B-face after learning to the A-face before learning, and (2) the similarity of the A-face after learning to the B-face before learning. We found such asymmetry in the left IFG only in the PK pair type, suggesting assimilation of new information into prior knowledge structures (Results, main text). It is informative to examine each of these values separately, to ascertain that indeed the difference stems from positive similarity between the B-face after learning to the A-face before learning, the direction that is consistent with our interpretation (note that each of these similarity values were computed across the pre-learning and post-learning phases, mitigating the concern regarding autocorrelations between regressors in this analysis). As can be seen in Supplementary Figure 4, indeed the reported asymmetry stemmed from positive similarity between the B-face after learning and the A-face before learning. In PK pairs, this similarity value for paired faces was significantly different from zero ($t_{(1,18)} = 3.11$, $p = .006$) or from the same measure calculated for shuffled faces baseline (same faces, but shuffled to compute the similarity such that they are paired with the faces they did not appear with during the associative learning task, see Methods, main text; $t_{(1,18)} = 3.15$, $p = .006$). In contrast, the similarity between the A-face after learning and the B-face before learning did not differ from zero or shuffled faces ($t_{(1,18)}$'s < 1.65, $p > .11$). The difference between the two similarity measures that are used to compute the asymmetry measure (i.e., the similarity between the B-face after learning and the A-face before learning, and the similarity between the A-face after learning and the B-face before learning) was further significant for paired faces ($t_{(1,18)} = 2.71$, $p = .01$), but not for shuffled faces ($t_{(1,18)} = 1.69$, $p = .11$). When examining n-PK pairs, none of the above comparisons reached significance ($t_{(1,18)}$'s < 1.33, $p > .20$).

Supplementary Note 7. Asymmetry for remembered and forgotten pairs.

Left anterior hippocampus. In the main text, we report changes in similarity from before to after learning based on whether pairs belonged to PK or n-PK pairs, as well as whether they were

later remembered (high-confidence hits) or forgotten. For completeness, we examined also if asymmetry in the direction of changes. The asymmetry measure was computed as was done in the main analysis, in the left IFG, only separately for remembered and forgotten pairs. That is, we computed two similarity values: (1) the similarity of the second face in a pair (B-face) after learning, to the first face in the pair (A-face), and (2) the similarity of the A-face after learning to the B-face before learning, and subtracting the latter from the former (see main text, Methods and Results, for more details). We found no evidence for asymmetry in learning in the left anterior hippocampus. The asymmetry values for PK or n-PK pairs, either remembered or forgotten, did not differ from zero or from each other (Remembered: PK: M = .004 , SD = .10, n-PK: M = .025 , SD = .15; Forgotten: PK: M = .008 , SD = .11, n-PK: M = .028 , SD = .17; all $t'_{(17)} <$ 1.64, p's > .11).

Left inferior frontal gyrus. In the left inferior frontal gyrus, we found asymmetry in representational changes for PK pairs that were associated together during learning compared to shuffled pairs (Results, main text). We examined asymmetry separately for remembered and forgotten pairs. We found no evidence for differences in asymmetry based on memory, as the asymmetry values for PK or n-PK pairs, either remembered or forgotten, did not significantly differ from zero or from each other (Remembered: PK: M = .002 , SD = .08, n-PK: M = -.007 , SD = .09; Forgotten: PK: M = .03, SD = .07, n-PK: M = .01, SD = .07; all $t'\text{s}_{(17)} < 1$, p's > .33, but Forgotten PK difference from zero: $t_{(17)} = 1.92$, $p = .072$).

Supplementary Note 8 (related to Supplementary Figure 5). Representational similarity in additional hippocampal ROIs. In the main text, we report the similarity changes in the left anterior hippocampus. Here we provide data from other hippocampal ROIs, namely, the right and left posterior hippocampus, and the right anterior hippocampus (Supplementary Figure 5). As in the left anterior hippocampus, similarity differences (post-learning minus pre-learning) in each ROI were submitted to a 2 (Prior Knowledge: PK, n-PK) by 2 (Memory: remembered – high-confidence hits only, forgotten) repeated-measures ANOVA. No main effects (Prior Knowledge/Memory) nor an interaction of Prior Knowledge by Memory were observed in the left posterior hippocampus, or in the right anterior hippocampus (all $F_{(1,17)}$'s < .67, p's > .42). In

the right posterior hippocampus, only the main effect of Prior Knowledge approached significance ($F_{(1,17)} < 2.82$, $p = .11$; the main effect of Memory and the interaction: $F_{(1,17)}$'s < .88, p's > .36). None of the simple comparisons (remembered PK vs. n-PK, or remembered vs. forgotten within each pair type) was significant ($t_{(17)}$'s < 1.34, p's > .19).

Supplementary Note 9 (related to Supplementary Figure 6). Representational similarity in additional cortical ROIs. In the main text, we report a number of regions that demonstrated higher functional connectivity for PK pairs compared to n-PK pairs (main text, and above, Supplementary Table 1). Of these, we focused on the left inferior frontal gyrus (left IFG) and the angular gyrus (AG), due to prior literature (see main text). As an exploratory analysis, we examined whether the additional cortical regions that demonstrated functional connectivity with the hippocampus showed asymmetry in the representational changes, as was observed in the left IFG (the left IFG and the AG are reported in the main text and are not repeated here). As in the left IFG and AG, a 12mm sphere was constructed around the peak voxel of that ROI in each participant's native space (note that for the right inferior frontal gyrus, the first peak reported here was at the edge of the brain, thus we constructed the sphere around another peak in that ROI, MNI coordinates: [50,30,-14]).

The data from these additional ROIs is presented in Supplementary Figure 6. Like in the left IFG analysis, prior to testing asymmetry in representational changes, we examined whether these regions showed any difference in similarity from pre-learning to post-learning. To briefly describe the analysis again here (see main text, Methods and Results, for more details), we computed the similarity between multivoxel activity patterns corresponding to the faces that where paired together during the associative learning task. These similarity values are computed before and after learning and a difference score is calculated by subtracting the former from the later. We then average these values for PK pairs and n-PK pairs. We further compare the similarity between paired faces to the similarity between shuffled-pairs, namely faces that did not appear together in the associative learning task. These shuffled pairs serve as baseline. The similarity differences (from pre to post) in each ROI were submitted to a repeated-measures ANOVA of Prior Knowledge (PK/n-PK) by Pairing (paired/shuffled). In

similarity changes from before to after learning, the left middle temporal gyrus (left MTG) and the left orbitofrontal cortex (left OFC) revealed a similar IFG (albeit statistically weaker) pattern to the left, namely, higher similarity for paired faces versus shuffled faces, without an interaction with Prior Knowledge (Pairing main effect: left MTG: $F_{(1,18)} = 5.67$, $p = .029$, left OFC: $F_{(1,18)} = 4.13$, $p = .057$; main effects of Prior Knowledge and interactions: $F_{(1,18)}$'s < .56, $p > .46$). The simple effects of paired vs. shuffled faces within each Prior Knowledge pair type did not reach significance (left MTG: PK: $t_{(1,18)} = 1.39$, $p = .18$; n-PK: $t_{(1,18)} = 1.96$, $p = .07$; left OFC: PK: $t_{(1,18)} = 1.64$, $p = .12$; n-PK: $t_{(1,18)} = 1.27$, $p = .22$). Of the other ROIs, in the right inferior frontal gyrus (right IFG) we observed a marginal main effect of Prior Knowledge ($F_{(1,18)} = 3.19$, $p = .09$; main effect of Pairing, or interaction of Pairing by Prior Knowledge, $F_{(1,18)}$'s < 2.44, $p > .13$), driven mostly by higher similarity differences for paired n-PK faces (paired vs. shuffled: n-PK: $t_{(1,18)} = 2.18$, $p = .04$; PK: $t_{(1,18)} = .30$, $p = .77$). The other ROIs (dorsomedial prefrontal cortex, right superior frontal gyrus, left inferior frontal gyrus, dorsal portion) did not exhibit any main effect nor an interaction of Prior Knowledge by Pairing ($F_{(1,18)}$'s < 2.00, $p > .17$; simple effects of paired vs. shuffled: $t_{(1,18)}$'s < 1.19, p's > .25).

Interestingly, when examining asymmetry in learning, no region has shown the pattern demonstrated by the left IFG. Asymmetry in learning was calculated by computing two similarity values: (1) the similarity of the second face in a pair (B-face) after learning, to the first face in the pair (A-face), and (2) the similarity of the A-face after learning to the B-face before learning, and subtracting the latter from the former. A positive value would mean that the neural representation of the B-face became similar to the A's face representation during learning (see main text, Methods and Results, for more details). In the main text, we report such positive asymmetry in learning in the left IFG only in PK pairs, suggesting assimilation of new information into prior knowledge structures. As can be seen in Supplementary Figure 6, no other region showed this pattern. In fact, some regions showed a negative asymmetry value, meaning that the A-face after learning became similar to the B-face before learning (more so than vice-versa). While a couple of regions showed qualitatively negative asymmetry for PK pairs, asymmetry was significantly different for paired compared to shuffled pairs in n-PK pairs in three ROIS (dorsomedial prefrontal cortex, right superior frontal gyrus, and right IFG: $t_{(18)}$'s >

2.2, p's < .05, in these regions asymmetry for n-PK paired faces was also significantly different from zero $t_{(18)}$'s > 2.50, p's < .03). These negative asymmetry values might suggest prediction of the B-face upon seeing the A-face[4]. However, note that while in the right IFG asymmetry was found along with a significant increase in similarity from before to after learning in n-PK pairs, the right superior frontal gyrus did not demonstrate such an increase in similarity, and the dorsomedial prefrontal cortex only showed some minor qualitative increase in similarity from before to after learning. Thus, the asymmetry values in these regions should be interpreted with cautious.

We now turn to similarity changes from before to after learning specifically for remembered versus forgotten pairs. In the left IFG, we did not observe a significant difference in similarity values based on memory. We did find such differences in the left anterior hippocampus (see Results, main text). The similarity changes from before to after learning in each of the additional ROIs were submitted to repeated-measures ANOVAs with the factors of Prior Knowledge (PK/n-PK) and Memory (Remembered, only high-confidence, as in the main analysis/Forgotten; see main text, Methods and Results). No significant main effects nor interactions were found in any of these ROI ($F_{(1,17)}$'s < 2.56, p > .12).

Supplementary Note 10. Representational changes for famous faces from pre-learning to post-learning in the hippocampus versus the left inferior frontal gyrus. In the main analysis in the hippocampus, we compared similarity between pairs of faces before and after learning. We found that the neural representations of famous and novel faces became more distinct, or more separated, from each other, through learning. An additional possible way to examine pattern separation in the hippocampus is to compute the similarity between the multivoxel activity pattern corresponding to a famous face prior to the associative learning after learning and compare that similarity value in the hippocampus to the same value in the left inferior frontal gyrus (left IFG). Note that this way of calculating similarity does not address separation between the famous face and its novel associated face, which was the focus of the current study. Nonetheless, it might be that the similarity of the same famous face from before to after

learning would be lower in the hippocampus, due to pattern separation, compared to that in the left IFG.

To that end, we computed, for each famous face, the similarity between the multivoxel activity pattern before and after learning, in the left anterior hippocampus (where we found the main result) and in the left IFG. We took only remembered pairs, as these are the pairs that showed separation in the left anterior hippocampus (see Main text). We found that the similarity of famous faces from before to after learning was indeed lower in the left anterior hippocampus compared to the left IFG (Hippocampus: $M = .003$, $SD = .09$; left IFG: $M = .017$, $SD = .05$). However, as can be seen in the SDs, there was large variance in the data, and the qualitative difference did not reach statistical significance ($t_{(17)} = .62$, $p = .52$). It is possible that these values are noisier than our main analysis due to the time that has passed between the pre-learning and the post-learning scans (in which participants were doing the associative learning task). In the hippocampal results reported in the main text, similarity is computed between activity patterns that were obtained in the same phase, i.e., similarity is computed between faces in the pre-learning phase, and between faces in the post-learning, and then the difference in similarity is computed. Another possibility is that the separation we see in the hippocampus between a famous face and its associated novel face is a result of both the famous face and the novel face becoming distinct from one another, and thus examining only the famous face leads to statistically weaker results. This suggestion is consistent with the lack of asymmetry in hippocampal separation, as reported above (Supplementary Note 7). Thus, while the results of this analysis are generally consistent with our main findings, the specific manner by which prior knowledge promotes hippocampal separation is a topic for future investigation.

Supplementary note 11 (related to Supplementary Table 2). We note that this study was not designed to address univariate differences between PK and n-PK pairs. Univariate effects are susceptible to repetition suppression (a reduction in univariate activation due to repetition). We likely had repetition suppression within trials, since we included two rapid presentations of each face in the pair in each trial. Additionally, each double-presentation of the pair repeated in

the study 12 times (in 12 cycles), which lead to further suppression across repetitions. (See Methods, Main text).

Even though the study was not designed for this purpose and thus we do not interpret these findings, for completeness, we report univariate activation during the associative learning task. To that end, we fit a GLM which included a regressor per pair type (12 PK pairs, 12 n-PK pairs) and repetition (we repeated the pairs 12 times during the study, hence, 24 regressors). We then ran a whole brain analysis comparing univariate activity in the first presentation of PK vs. n-PK pairs. In this contrast we sacrificed power in favor of avoiding repetition suppression across trials. This analysis did not reveal any significant cluster for PK > n-PK or the opposite contrast (thresholding of .001, voxel level, and a cluster size of 50 contingent voxels to correct for multiple comparisons at $p < .05$, determined by Monte-Carlo simulation). In a more lenient threshold (voxel level: $p < .005$), we find a cluster in the ventromedial prefrontal cortex (vmPFC) demonstrating higher activation for PK compared to n-PK pairs, and a precuneus cluster demonstrating higher activation for n-PK compared to PK pairs (Supplementary Table 2; a cluster of 61 contingent voxels corrects for multiple-comparisons).

We additionally examined the comparison between PK and n-PK pairs across all repetitions. This analysis maximizes power, and likely reflects repetition suppression. A few regions including the middle temporal gyrus and the middle frontal gyrus revealed higher activation for n-PK pairs compared to PK pairs, potentially reflecting larger repetition suppression for PK pairs (Supplementary Table 2). No region showed higher activation for PK compared to n-PK pairs, also in the more lenient threshold (as above).

References

1. Bates, D., Mächler, M., Bolker, B. & Walker, S. Fitting Linear Mixed-Effects Models using lme4. *J. Stat. Softw.* **67**, (2015).
2. Lo, S. & Andrews, S. To transform or not to transform : using generalized linear mixed models to analyse reaction time data. **6**, 1–16 (2015).
3. Kim, G., Norman, K. A. & Turk-Browne, N. B. Neural differentiation of incorrectly predicted memories. *J. Neurosci.* **37**, 2022–2031 (2017).
4. Schapiro, A. C., Kustner, L. V & Turk-Browne, N. B. Shaping of Object Representations in the Human Medial Temporal Lobe Based on Temporal Regularities -supplementary

information. *Curr. Biol.* **22**, 1622–1627 (2012).
5. Schlichting, M. L., Mumford, J. A. & Preston, A. R. Learning-related representational changes reveal dissociable integration and separation signatures in the hippocampus and prefrontal cortex. *Nat. Commun.* **6**, 1–10 (2015).
6. Mumford, J. A., Turner, B. O., Ashby, F. G. & Poldrack, R. A. Deconvolving BOLD activation in event-related designs for multivoxel pattern classification analyses. *Neuroimage* **59**, 2636–2643 (2012).
7. Mumford, J. A., Davis, T. & Poldrack, R. A. The impact of study design on pattern estimation for single-trial multivariate pattern analysis. *Neuroimage* **103**, 130–138 (2014).
8. Dragoi, G. & Tonegawa, S. Preplay of future place cell sequences by hippocampal cellular assemblies. *Nature* **469**, 397–401 (2011).
9. Jafarpour, A., Piai, V., Lin, J. J. & Knight, R. T. Human hippocampal pre-activation predicts behavior. *Sci. Rep.* 1–9 (2017).
10. Sadeh, T., Chen, J., Goshen-Gottstein, Y. & Moscovitch, M. Overlap between hippocampal pre-encoding and encoding patterns supports episodic memory. *Hippocampus* **29**, 836–847 (2019).
