# Supplementary material for: Prior knowledge promotes hippocampal separation but cortical assimilation in the left inferior frontal gyrus
Source: Nat Commun. 2020 Sep 14;11:4590. doi: 10.1038/s41467-020-18364-1 (PMC7490707; doi:10.1038/s41467-020-18364-1)
Supplement: Supplementary file 3 — Reporting Summary [file 41467_2020_18364_MOESM3_ESM.pdf]

nature research

Corresponding author(s): Anat Maril

Last updated by author(s): Jul 18, 2020

Reporting Summary

Nature Research wishes to improve the reproducibility of the work that we publish. This form provides structure for consistency and transparency in reporting. For further information on Nature Research policies, see Authors & Referees and the Editorial Policy Checklist.

Statistics

For all statistical analyses, confirm that the following items are present in the figure legend, table legend, main text, or Methods section.

n/a	Confirmed	
☐	☒	The exact sample size (n) for each experimental group/condition, given as a discrete number and unit of measurement
☐	☒	A statement on whether measurements were taken from distinct samples or whether the same sample was measured repeatedly
☐	☒	The statistical test(s) used AND whether they are one- or two-sided<br>*Only common tests should be described solely by name; describe more complex techniques in the Methods section.*
☐	☒	A description of all covariates tested
☐	☒	A description of any assumptions or corrections, such as tests of normality and adjustment for multiple comparisons
☐	☒	A full description of the statistical parameters including central tendency (e.g. means) or other basic estimates (e.g. regression coefficient) AND variation (e.g. standard deviation) or associated estimates of uncertainty (e.g. confidence intervals)
☐	☒	For null hypothesis testing, the test statistic (e.g. F, t, r) with confidence intervals, effect sizes, degrees of freedom and P value noted<br>*Give P values as exact values whenever suitable.*
☒	☐	For Bayesian analysis, information on the choice of priors and Markov chain Monte Carlo settings
☐	☒	For hierarchical and complex designs, identification of the appropriate level for tests and full reporting of outcomes
☐	☒	Estimates of effect sizes (e.g. Cohen's d, Pearson's r), indicating how they were calculated

Our web collection on statistics for biologists contains articles on many of the points above.

Software and code

Policy information about availability of computer code

Data collection	MATLAB R2018b; optseq2, https://surfer.nmr.mgh.harvard.edu/optseq/; Presentation software 16.5, Neurobehavioral Systems, Inc., Berkeley,CA, www.neurobs.com
Data analysis	SPM8; MATLAB R2018b; FSL 5.0.2.2 (FMRIB; Smith et al., 2004); R version 3.5.2 (R Core Team, 2018); Lme4 (Bates et al., 2014); costume code is available at https://github.com/odedbein/SEL_public.

For manuscripts utilizing custom algorithms or software that are central to the research but not yet described in published literature, software must be made available to editors/reviewers. We strongly encourage code deposition in a community repository (e.g. GitHub). See the Nature Research guidelines for submitting code & software for further information.

Data

Policy information about availability of data

All manuscripts must include a data availability statement. This statement should provide the following information, where applicable:

- Accession codes, unique identifiers, or web links for publicly available datasets
- A list of figures that have associated raw data
- A description of any restrictions on data availability

Raw data and single-trial t-statistic maps that support the findings of this study are available on https://osf.io/u2h3s/. Additional data will be provided from the corresponding author upon a reasonable request.

Field-specific reporting

Please select the one below that is the best fit for your research. If you are not sure, read the appropriate sections before making your selection.

☒ Life sciences ☐ Behavioural & social sciences ☐ Ecological, evolutionary & environmental sciences

For a reference copy of the document with all sections, see nature.com/documents/nr-reporting-summary-flat.pdf

Life sciences study design

All studies must disclose on these points even when the disclosure is negative.

Sample size	Twenty-four participants were scanned in this study (19 included in the final sample). This sample size was determined based on similar previous studies, and taking into account possible exclusions (Reggev et al., 2016, N = 19; Bein et al., 2014, N = 20; Schapiro et al., 2012, N = 17).
Data exclusions	Five participants were excluded from the analysis: two due to excessive movement (more than 3mm across all pre-learning, post-learning, and associative learning scans); two due to insufficient knowledge about the famous faces, as defined by familiarity with fewer than two thirds of the faces in a post-experiment questionnaire; and one due to poor compliance with the task instructions leading to lower than chance performance in the final memory test
Replication	We do not offer a replication, this is a single fMRI study. Future research should aim to replicate our findings.
Randomization	The experiment was designed as a within-participant study, thus all participants participated in all experimental conditions.
Blinding	Blinding was irrelevant, as this was a within-participant study.

Reporting for specific materials, systems and methods

We require information from authors about some types of materials, experimental systems and methods used in many studies. Here, indicate whether each material, system or method listed is relevant to your study. If you are not sure if a list item applies to your research, read the appropriate section before selecting a response.

Materials & experimental systems

n/a	Involved in the study	
☒	☐	Antibodies
☒	☐	Eukaryotic cell lines
☒	☐	Palaeontology
☒	☐	Animals and other organisms
☐	☒	Human research participants
☒	☐	Clinical data

Methods

n/a	Involved in the study	
☒	☐	ChIP-seq
☒	☐	Flow cytometry
☐	☒	MRI-based neuroimaging

Human research participants

Policy information about studies involving human research participants

Population characteristics	Participants were native Hebrew-speakers between the ages of 19-35 (in the eventual sample, we had nine women; mean age: 26.94 years, range: 22-31 years). Participants were further screened for having normal or corrected to normal vision and no color-blindness and to ensure they had no neurological conditions or any other contraindications for MRI (based on self-report).
Recruitment	Participants were recruited based on fliers and adds from the Hebrew University of Jerusalem community. Bias in the selection is unlikely, as this is a within-participant design. All measures were taken within each individual participant.
Ethics oversight	The Tel Aviv Sorasky Medical Center Ethics Committee and The Hebrew University institutional review board

Note that full information on the approval of the study protocol must also be provided in the manuscript.

Magnetic resonance imaging

Experimental design

Design type	event-related
Design specifications	Per participant, we had 12 pairs in each condition (Prior Knowledge, No Prior Knowledge, total of 24 pairs per

Design specifications	participant). This allow meaningful representational similarity analysis between items in the pairs, in line with previous similar studies (Schapiro et al., 2012, 2 pairs; Schlichting et al., 2015, 6 pairs). During the pre-learning and post-learning scans (for our main analyses), each item was presented for 1 s. Trials were jittered with .5-7.5 s of a fixation-cross baseline, with an interval of .5 s, using optseq2 (https://surfer.nmr.mgh.harvard.edu/optseq/; Dale, 1999). During the associative-learning task, each trial lasted 3 -s. Again, trials were further jittered with .5-7.5 s fixation-cross baseline, with an interval of .5 s (Dale, 1999).
Behavioral performance measures	The mean accuracy and reaction times are reported for the associative learning task (the spread of the reaction times is presented in a figure). Mean accuracy during the pre- and post-learning scans is reported as well. Participants were highly accurate in both tasks (mean > 96%). For the associative memory test, we report the mean and standard deviation of overall accuracy and confidence rates. Accuracy rates were significantly different from chance, indicating memory of the learned pairs.

Acquisition

Imaging type(s)	functional and structural
Field strength	3T
Sequence & imaging parameters	Participants were scanned in a 3T Simens Prisma scanner. The experiment included an MPRAGE anatomical scan (1X1X1mm resolution), a fieldmap scan, and 12 whole-brain T2*-weighted EPI scans (TR=2000 ms, 200-mm*180mm FOV, 64x58 matrix, TE=28, flip angle=77, phase encoding direction: anterior-posterior). In each volume, 39 slices were acquired tilted minus 20 degrees of the AC-PC, 3.125*3.125*3.1-mm (width*length*thickness) voxel size, no gap, in a top-down interleaved order. In each of the four sessions of the pre-post task, 366 images were acquired. Each of the four sessions of the learning task included 179 images.
Area of acquisition	whole brain

Diffusion MRI ☐ Used ☒ Not used

Preprocessing

Preprocessing software	SPM 8
Normalization	The pre-learning and post-learning representational similarity analysis was conducted in the native participants-space to avoid smoothing and normalization (Kriegeskorte, 2008). The associative learning data was normalized to MNI space using non-linear transformations.
Normalization template	The pre-learning and post-learning representational similarity analysis was conducted in the native participants-space to avoid smoothing and normalization (Kriegeskorte, 2008). The associative learning data was normalized to MNI space using non-linear transformations.
Noise and artifact removal	SPM 8 default motion correction and slice-timing correction was applied
Volume censoring	none

Statistical modeling & inference

Model type and settings	RSA: item-specific t-statistics were estimated based on first-level fixed effects models. Average similarity was computed per participant and condition, and entered to group level statistics. PPI: the results from first-level fixed effects models were then entered to a second-level group analysis, using participants as random effects.
Effect(s) tested	RSA, similarity differences from pre- to post-learning: a repeated-measures 2 by 2 ANOVA of memory (high-confidence hits, misses) by Pair Type (Prior Knowledge vs. no-Prior Knowledge). T-tests were used to test pairwise simple effects between memory levels within Pair Type, and to compare similarity of high-confidence hits to shuffled pairs. Paired sample t-tests were also used to test for significance in our asymmetry measure, either against shuffled pairs or 0.

Specify type of analysis: ☐ Whole brain ☐ ROI-based ☒ Both

Anatomical location(s)	The hippocampus was anatomically defined using FSL's FIRST automated anatomical segmentation. The division to anterior and posterior portion was made based on the most anterior or posterior third of the coronal slices, respectively, by an in-house MATLAB script. the left inferior frontal gyrus ROI was functionally defined based on the PPI analysis.
Statistic type for inference (See Eklund et al. 2016)	voxel-level of p < .005 was used due to low power in PPI designs (O'Reilly, Woolrich, Behrens, Smith, & Johansen-Berg, 2012), accounting for the reduced voxel-level threshold by maintaining a cluster level threshold of p < .05 (Brod et al., 2016; resulting in cluster size > 61 voxels, Monte Carlo simulations, Slotnick, Moo, Segal, & Hart, 2003).
Correction	Monte Carlo simulations (Slotnick, Moo, Segal, & Hart, 2003)

Models & analysis

n/a	Involved in the study
☐	☒ Functional and/or effective connectivity
☒	☐ Graph analysis
☒	☐ Multivariate modeling or predictive analysis

Functional and/or effective connectivity	gPPI; implemented by SPM8 gPPI toolbox (McLaren et al., 2012)
