# Supplementary material for: Prior knowledge promotes hippocampal separation but cortical assimilation in the left inferior frontal gyrus
Source: Nat Commun. 2020 Sep 14;11:4590. doi: 10.1038/s41467-020-18364-1 (PMC7490707; doi:10.1038/s41467-020-18364-1)
Supplement: Supplementary file 2 — Peer Review File [file 41467_2020_18364_MOESM2_ESM.docx]

Peer Review Information

**Journal:** Nature Communications

**Manuscript title:** Prior knowledge promotes hippocampal separation but cortical assimilation in the left inferior frontal gyrus

**Corresponding author name(s):** Pro Anat Maril

**Editorial notes:**

**none**

**Reviewer comments & decisions:**

**Reviewer comments, first version:**

Reviewers' comments:

Reviewer #1 (Remarks to the Author):

This paper assesses how changes in hippocampal and cortical representations are shaped by prior knowledge. Participants in an fMRI study learned pairs of faces which either did or did not include a famous face. From before to after learning, the representations of pairs of famous and unknown faces differentiated in the hippocampus but merged in the neocortex, with the novel face becoming more similar to the known face. Pairs of unknown faces became more similar in the hippocampus.

Both the findings of a dissociation between integration and separation as a function of prior knowledge within the hippocampus and the representational assimilation in cortex are very exciting discoveries. I felt that the study was well designed and the methods were rigorous, and I am overall enthusiastic.

My biggest concern is that there was some selective reporting and displaying of results, which makes the results appear more straightforward. For example, the authors tested left and right hippocampus, and anterior and posterior segments, but they only reported and displayed the results for the left anterior hippocampus (in Fig 2). The results should be reported and displayed in full. It also felt strange to focus on lIFG for the analyses of cortical changes in similarity. The authors likely looked at the other regions showing significant connectivity with the hippocampus, in which case these results should be reported. As an additional side issue, there are no stats reported for the lIFG changes in similarity.

It would be useful to see the raw initial and final similarity for each condition in the hippocampus in addition to the change scores, perhaps as a supplemental figure. The interpretation of change scores can vary as a function of initial values.

I may have missed this, but I’m not sure the authors ran a simple contrast of univariate activity differences between PK and nPK, which would be useful to report.

I puzzled over the claim in a couple places that RSA but not univariate/connectivity methods provides information about “content”. Maybe this claim could be unpacked. RSA does provide information that is specific to individual items, but what endows this method in particular with the ability to see “content”? Similarly, in the Discussion, the authors claim that this study, unlike prior ones, can address what “learning process” is taking place. I’m not sure about this as a qualitative distinction. This study shows representational changes that could be a result of many different kinds of learning processes. Perhaps RSA is a more constraining kind of measurement, but I think the claim that it provides special access to content and learning mechanisms would need some defending.

Minor: In the supplement, line 65, there is a sentence that ends prematurely.


Reviewer #2 (Remarks to the Author):

Summary of Paper

This paper had two main hypotheses: (1) when a novel item is being associated to a familiar item (existing knowledge), the cortical system is biased towards assimilation whereas the hippocampus is biased towards pattern separation to prevent interference; (2) learning this association results in asymmetric cortical effects in that novel items, compared to familiar items, will go through relatively larger transformations. Using fMRI, the researchers found evidence that supported their hypotheses: (1) multivoxel activity patterns representing familiar-novel pairs became separated in the hippocampus whereas the novel item in the novel-familiar pair became more similar (i.e. integrated) to the familiar item after learning, and (2) positive asymmetry was found for famous-novel pairs but not for novel-novel pairs. Altogether, these findings support the influence of existing knowledge on learning.

Summary of Comments

The direction of assimilation and the observed asymmetric cortical effects are exciting findings. While the study contributes important additions to the literature, the paper requires substantial edits to enhance clarity of methodology and logic in the analyses conducted.

Major Comments

For ease of commenting, I refer to:
● F1 as the pre-learning famous face
● F2 as the post-learning famous face
● N1 as the pre-learning novel face that is eventually paired with the famous face
● N2 as the post-learning novel face that was paired with the famous face
● r is representational similarity

1. While the current study computed r(F2,N2) - r(F1,N1) to provide evidence for pattern separation, one also can assess pattern separation in another way: assuming that F2 changes with learning, one could compare 1) the change in representation of the famous face in the hippocampus (F2 in HPC - F1 in HPC) to 2) the change in representation of the famous face in the cortex (F2 in cortex - F1 in cortex). If pattern separation is occurring in HPC and assimilation in the cortex, 1) would be larger than 2). It may be worthwhile to conduct this analysis to support your current evidence.

2. When assessing whether N2 has become integrated with the existing representation of the famous face, the authors assume that this integration occurs between F1 and N2, rather than F2 and N2. (i.e. to assess asymmetric cortical updating they calculate the similarity between the representation of a novel face after learning to the original representation of the famous face with which it was associated.) Why is it assumed that the novel face becomes assimilated into the original face representation, rather than the updated face representation (F2)? In real-time integration, the concept space may become modified with the addition of a new item. Integration of the novel face in the existing face representation may co-occur with the updating of the existing face representation, thus resulting in N2 becoming integrated with F2 rather than F1.

Minor Comments

1. Line 111 – The idea that existing representations are stronger is one that hasn’t been introduced beforehand; this assumes the reader knows what makes a representation “stronger” than another.
2. Line 180, line 301 – the expectation for increased similarity in hippocampus for novel items was not set up in the Introduction and comes across as an unexpected finding.
3. Line 838 – beyond pixel correlations, were other details controlled for such as emotional valence or hair length?
4. Lines 841-842 require rewording to clarify the methodology. Perhaps replace with “2 B-faces were paired with each A-face pair. These pairings were random for each participant, although they were fixed within the experiment…”
5. Line 874 – What was the purpose of this “irrelevant task”?
6. Lines 891-895 require clarification because there is a jump in the logic. What do you mean by “regressor of that face”? This concept was inadequately introduced/explained.
7. Line 940 – aren’t the three faces that appear at the bottom of the screen 2 B-faces and 1 A-face (the other item in the A-face pair?) If all novel faces are supposed to be B-faces (even the one that was paired to the famous face), then lines 841-842 need to be revised.

Line-by-Line Edits

1. Line 24 – remove “together”
2. Line 28 – remove the hyphen between “highly” and “familiar”
3. Line 44 – switch the order of “media posts” and “ads” to parallel logic of “…new album or starts dating…”
4. Line 45 – move towards active voice rather than passive. E.g. “How do we form such new associations?”
5. Line 63 – after “lovers” change n-dash to m-dash
6. Line 130 – “post-learning” instead of “post-similarity”
7. Line 277 – “how individual associations are…” to parallel previous structure
8. Line 286 – remove “critically” -- too repetitive
9. Line 308 – colon instead of comma
10. Line 388 – remove “asking” and rephrase sentence
11. Line 815 – add hyphen in “two-thirds”
12. Line 848 – choose “by”, “x” or “*” for describing resolution and keep consistent
13. Lines 872-873 – restructure sentence to move emphasis off of testing and onto the post-learning scan. E.g. “Critically, acquiring the post-learning scans before testing memory allowed us to measure post-learning representations without interference…”
14. Line 973 – add “e” to Siemens
15. Line 978 – add “/” instead of hyphen in pre/post


Figure Edits

1. Figure 1.d) – “confidence”


Reviewer #3 (Remarks to the Author):

Bein and colleagues report and fMRI study in which they manipulated the extent to which new associations could be incorporated into prior knowledge. They accomplished this by having half of the pairs include one famous (e.g., Madonna) and one novel face (PK pairs); the other pairs were two novel faces (n-PK pairs). They found that the representation of the two paired faces changed from pre- to post- learning in a way that was dependent on the prior knowledge manipulation: in anterior hippocampus, items in PK pairs became less similar with learning, while n-PK pairs became more similar. The same anterior hippocampal region showed greater connectivity with a network of neocortical regions (including LIFG) specifically during PK encoding. LIFG also showed an increase for the PK pairs which was driven by the novel face coming to be represented more similarly to the famous face ("assimilation").

I am generally enthusiastic about the topic and this paper. The manuscript itself is generally clear and well-written, and the results are very interesting. I do have some concerns and questions with respect to interpretation and methods, however.

Major comments:

1. To what extent are the findings about asymmetry specific to LIFG? First of all, the section is titled "cortex," which is extremely vague and borderline misleading, as the authors only report one of their (several) cortical regions. I understand the idea is to align this with CLS but the authors are not showing assimilation in "cortex," but rather just IFG. Please retitle this to be about LIFG if that is the only region that will be discussed. I think the paper title should be revised for the same reason. Second, why were the other functional regions (derived from the same analysis as the IFG region that was interrogated; shown in Figure 3) not tested? Perhaps it is specific to IFG (which would be interesting), but perhaps it is not (which in my mind is also interesting). Either way, I think it's important to know the answer to this.

2. I kept thinking to myself that the involvement of LIFG in language seems to be important here, since the famous faces critically differ from the non-famous faces in how nameable they are. Perhaps participants are bringing to mind the verbal label ("Madonna") and this is driving the apparent asymmetry. Do the authors suspect this is what is going on? To what extent is this an issue for the interpretation, if at all? I think the authors intend for the "Madonna" schema to be more than the verbal label itself. This feels like an important limitation of and discussion point for the study, and one that is especially true in the realm of face stimuli. (For example, both famous and non-famous locations/scenes are somewhat nameable, though albeit to different degrees or levels of specificity.)

3. I do not share the authors' intuition that asymmetry going in the B-->A direction cannot mean that B is not bringing to mind A (p. 19). It is true that it is in the opposite direction of the temporal relationship, but that is only one dimension that matters. (Also as I understand it, since each pair was repeated twice ABAB with no gap, that means that B does come before A?) Surely direction/ease of movement within semantic or associative networks need also be considered. Here, the A item is the (by design) more well-known stimulus; why would it be surprising that B would trigger retrieval of A? Certainly the ease and fidelity of reactivation of a well-known (famous) face would be expected to be higher (B-->A) than a novel face (A-->B). This is somewhat related to my point about the verbal labels (famous people's names).

4. Was there a relationship between the amount of dissimilarity in hippocampus and the amount of similarity in LIFG? Ideally the authors could ask this on a trialwise basis to determine whether hippocampal separation and LIFG assimilation are occurring together on the same set of trials (i.e., are positively related), or across different trials (unrelated or perhaps even negatively related). Knowing the nature of this relationship might change or strengthen the authors' interpretation.

5. Was there asymmetry in the movement of hippocampal representations? Or, why wasn't this investigated?

6. Since the analysis was performed for only those trials that were remembered, it would be important to demonstrate that the observed representational change differences are not due to differences in the number of trials and thus the stability of estimates between the conditions. This could be accomplished by subsampling the condition with the larger number of comparisons and re-performing the analyses.

7. It was not clear to me from reading the paper whether the change in representation was shown to be greater for PK vs. n-PK pairs (p. 8). This section says that there was change within the pairs, and that there was a difference between the paired items versus a shuffled baseline. However, this might be a difference observed only in the post-learning scan, rather than a difference in the amount of representational change. Please clarify and if it has not already been done, confirm that representational change is greater for paired items vs. the shuffled (within condition) control. (I believe the authors probably *have* done this, since that it how it is described later in the paper for a similar analysis.)

8. The authors' approach of averaging beta-values across all pre-learning scans is not ideal as inflated similarity of the signal within-run will influence the pattern similarity values (and might bias the analyses, though I think this would depend on the specific orders). See Mumford, Davis & Poldrack (2014). These analyses should be changed to adopt this (what I would call) field standard approach, and all comparisons (correlations) restricted to betas derived from different scanning runs. It should at the very least be demonstrated and included in the paper whether the results stay the same when the analysis is performed in this way.

Minor comments:

9. In the introduction, the authors make the case that prior knowledge might bias the hippocampus towards pattern separation (top of p. 5), but it is not clear to me why this would be the prediction. Couldn't it be just as easily predicted that the hippocampus pattern complete (e.g., to related associations and memories that exist for familiar but not novel faces)? Please clarify.

10. In the prior knowledge case, really only one thing needs to be learned (or maybe two; the new face + the link with the existing knowledge). To what extent is this driven by better, or more precise representation of the novel face that is part of the PK pairs? In other words, one might expect that since participants can spend more time encoding the novel face in these pairs, which could lead to a more specific representation of the items. Perhaps the authors have already performed an analysis that can rule this out.

11. Pattern similarity analyses were limited to correct (remembered) trials, whereas I believe PPI was performed across all trials. Please explain the reasoning behind this decision.

12. I assume the LIFG asymmetry analysis was performed only for remembered associations as well; please clarify.

13. Schlichting, Mumford & Preston (2015) found evidence for both separation and integration in the anterior hippocampus across different learning conditions, not integration overall as is implied on p. 16 of the discussion. The direction of representational change depended on the learning conditions, which might be useful to note in the present discussion (and related to the text in the immediately preceding paragraph).

14. At a couple of points in the paper the authors use the term "transitive inference" where I believe they mean "associative inference."

15. Were the faces in the n-PK condition on average matched in age with the PK pairs? I am wondering whether the same-age bias might mean the participants would have a better ability to represent the faces in the PK pairs if they are as whole closer in age to the participants than the n-PK stimuli.

**Author rebuttal, first version:**

Reviewer #1 (Remarks to the Author):

This paper assesses how changes in hippocampal and cortical representations are shaped by prior knowledge. Participants in an fMRI study learned pairs of faces which either did or did not include a famous face. From before to after learning, the representations of pairs of famous and unknown faces differentiated in the hippocampus but merged in the neocortex, with the novel face becoming more similar to the known face. Pairs of unknown faces became more similar in the hippocampus.

Both the findings of a dissociation between integration and separation as a function of prior knowledge within the hippocampus and the representational assimilation in cortex are very exciting discoveries. I felt that the study was well designed and the methods were rigorous, and I am overall enthusiastic.

**Thank you for the enthusiastic evaluation of our work.**

My biggest concern is that there was some selective reporting and displaying of results, which makes the results appear more straightforward. For example, the authors tested left and right hippocampus, and anterior and posterior segments, but they only reported and displayed the results for the left anterior hippocampus (in Fig 2). The results should be reported and displayed in full. It also felt strange to focus on lIFG for the analyses of cortical changes in similarity. The authors likely looked at the other regions showing significant connectivity with the hippocampus, in which case these results should be reported. As an additional side issue, there are no stats reported for the lIFG changes in similarity.

**We thank the reviewer for the comment, and regret not making our report of additional results clearer.**

**As a reminder, we examined changes in the similarity between multivoxel BOLD activity patterns corresponding to faces that were paired together during an associative learning task. In the left anterior hippocampus, we found an interaction of Prior Knowledge (PK pairs, including a famous face and a novel face, vs. n-PK pairs, including two novel faces) by Memory (remembered vs. forgotten in a subsequent associative memory test). The similarity between faces in remembered PK pairs decreased through learning, in contrast to increased similarity between faces in remembered n-PK pairs.**

**We indeed examined additional hippocampal ROIs and did not observe interactions nor main effects of Prior Knowledge or Memory. In the previous version of the manuscript, this was mentioned in a foot note. We now mention these data in the main text, after reporting the results from the left anterior hippocampus: *“No significant interactions nor main effects were observed elsewhere in the hippocampus. The data from the other hippocampal ROIs are reported in the Supplementary Information.”*** **(p. 8).**

**In the Supplementary Information we report the results in full. We added the following text and figure:**

***“In the main text, we report the similarity changes in the left anterior hippocampus. Here we provide data from other hippocampal ROIs, namely, the right and left posterior hippocampus, and the right anterior hippocampus (Figure S5). As in the left anterior hippocampus, similarity differences (post-learning minus pre-learning) in each ROI were submitted to a 2 (Prior Knowledge: PK, n-PK) by 2 (Memory: remembered – high-confidence hits only, forgotten) repeated-measures ANOVA. No main effects (Prior Knowledge/Memory) nor an interaction of Prior Knowledge by Memory were observed in the left posterior hippocampus, or in the right anterior hippocampus (all F_(1,17)_’s < .67, p’s > .42). In the right posterior hippocampus, only the main effect of Prior Knowledge approached significance (F_(1,17)_ < 2.82, p = .11; the main effect of Memory and the interaction: F_(1,17)_’s < .88, p’s > .36). None of the simple comparisons (remembered PK vs. n-PK, or remembered vs. forgotten within each pair type) was significant (t_(17)_’s < 1.34, p’s > .19).”***

*Figure S5*. Representational similarity changes in additional hippocampus ROIs. ΔSimilarity: similarity post-learning minus similarity pre-learning. PK: prior knowledge pairs, n-PK: no prior knowledge pairs. Error bars reflect +-1 SEM. The results from the left anterior hippocampus are reported in the main text.

**Regarding the data in cortical regions, in the left inferior frontal gyrus (left IFG), we found an increase in the representational similarity from before to after learning between pairs of faces that appeared together during the associative learning task. Critically, we additionally quantified the direction of the changes in similarity by computing an asymmetry measure. Asymmetry in learning was calculated by computing two similarity values: (1) the similarity of the second face in a pair (B-face) after learning, to the first face in the pair (A-face), and (2) the similarity of the A-face after learning to the B-face before learning, and subtracting the latter from the former. We found such asymmetry in the left IFG, only in PK pairs, suggesting that the novel B-face was assimilated into knowledge structures.**

**Regarding the overall changes in similarity from pre-learning to post-learning in the LIFG, we have previously reported these results in full in the supplemental and only described them in the main text. Following this reviewer and Reviewer #2’s comment, we have now moved this section to the main text, prior to reporting the main asymmetry result:**

***“Although we targeted asymmetric changes in the representational patterns from before to after learning, we first examined whether any changes in representational similarity occurred from before to after learning. To that end, we asked whether there was evidence of learning-related similarity changes from pre- to post-learning in the left IFG. For each pair type (PK/n-PK), we compared the change in representational similarity between pairs of faces that appeared together during the learning task to the shuffled pairs baseline (faces from the same PK/n-PK pair-type that did not appear together at learning, see Methods). This comparison allowed us to specifically examine changes due to associative learning, controlling for similarity differences due to item familiarity or type of pair (Schapiro et al. 2012). A repeated-measures ANOVA with Prior Knowledge (PK/n-PK) and Pairing (paired/shuffled), revealing a main effect of Pairing (F_(1,18)_ = 11.44, p = .003, η_p_^2^ = .32), and no main effect of Prior Knowledge or Prior Knowledge by Pairing interaction (F’s < 1.2, p’s > .29). Pairwise comparisons showed a highly significant increase in similarity in PK pairs (PK: paired: M = .03, SD = .04; shuffled: M = -.01, SD = .02; t_(18)_ = 4.1, p = .0007, Cohen’s d = .94). N-PK paired faces also became qualitatively more similar compared to shuffled baseline (paired: M = .02, SD = .06; shuffled: M = .00, SD = .02; t_(18)_ = 1.16, p = .26). Thus, we obtained greater increase in similarity specifically for paired faces, but not for shuffled faces, indicating that there was a change in similarity from before to after learning.”* (p. 12).**

**We have also modified the Methods section to include this type of similarity analysis (previously described in the supplementary):**

***“Learning-related pre-to-post similarity differences: Prior to examining asymmetry in representational changes (see Introduction, and below), it is important to know whether any change in similarity occurred from before to after learning. To that end we computed for each participant, and for each pair type (PK, n-PK), the average similarity difference between pre- and post-learning for pairs of items that appeared together during the learning task. As a control, we compared these similarity differences to shuffled pairs baseline. The shuffled pairs baseline was obtained by pairing each A-face with all other B-faces of the same pair type, computing the similarity differences for each pair, and averaging across all pairs in each pair type. Note that this within pair-type shuffling controls for any differences the B-faces may have due to their appearance with famous versus novel pairs, since for each condition, all faces in the shuffled baseline appeared with A-faces of the same pair type.”* (p. 41).**

**Accordingly, the section discussing pre to post-learning similarity changes in the left IFG was removed from the Supplementary Information.**

**Regarding our focus on the left IFG and additional cortical regions, as is mentioned in the manuscript (p. 11), we focused on the LIFG due to its known involvement in prior knowledge influences on new learning (Maril et al., 2011; Reggev et al., 2016; Staresina et al., 2009), as well as more broadly in semantics (Badre, Poldrack, Paré-Blagoev, Insler, & Wagner, 2005; Badre & Wagner, 2007; Binder & Desai, 2011). As we noted in a foot note in the previous version of the manuscript, we have additionally looked at the Angular Gyrus (AG), since it was recently proposed to bind aspects of schematic knowledge and mediating schema influences on encoding (Bein, Reggev, & Tompary, 2018; Gilboa & Marlatte, 2017; van der Linden, Berkers, Morris, & Fernández, 2017; I. C. Wagner et al., 2015). In the AG, we found no significant similarity changes from pre- to post-learning, with or without respect to subsequent memory (ANOVA’s of Prior Knowledge by Paired/Shuffled, or of Prior Knowledge by Memory, F’s < 2.08, p’s > .16). We thank the reviewer for pointing out that this was not clear enough in our previous version of the manuscript. We now added the results from the AG to the main text: “*We further examined whether asymmetry in learning was observed in other cortical regions that demonstrated functional connectivity with the left anterior hippocampus. We chose the angular gyrus (AG), since it was recently proposed to bind aspects of schematic knowledge and mediate schema influences on encoding (Bein, Reggev, & Tompary, 2018; Gilboa & Marlatte, 2017; van der Linden, Berkers, Morris, & Fernández, 2017; I. C. Wagner et al., 2015). In the AG, we found no similarity differences from pre- to post-learning, with or without respect to subsequent memory (ANOVA’s of Prior Knowledge by Paired/Shuffled, or of Prior Knowledge by Memory, F’s < 2.08, p’s > .16). For completeness, we have directly targeted asymmetry, which did not differ from zero or shuffled pairs in either PK or n-PK pairs (all t_(18)_’s < 1.46, p’s > .16).”* (p. 13).**

**Following this reviewer and Reviewer #3’s recommendation, as an exploratory analysis, we have now additionally examined additional cortical regions showing connectivity with the hippocampus. We report these results in full in the Supplementary Information and refer to them in the main text. In the main text, we added: *“As an exploratory analysis, we have examined the other cortical regions that demonstrated functional connectivity with the left anterior hippocampus. The data from these regions are reported in the Supplementary Information. Interestingly, even though some of the regions showed an overall increase in similarity from before to after learning, no other region that we examined has shown asymmetry in the direction of representational changes, as was observed in the left IFG. “* (p. 14).**

**In the Supplementary Information, we now added the following text and figure:**

***“In the main text, we report a number of regions that demonstrated higher functional connectivity for PK pairs compared to n-PK pairs (main text, and above, Table S1). Of these, we focused on the left inferior frontal gyrus (left IFG) and the angular gyrus (AG), due to prior literature (see main text). As an exploratory analysis, we examined whether the additional cortical regions that demonstrated functional connectivity with the hippocampus showed asymmetry in the representational changes, as was observed in the left IFG (the left IFG and the AG are reported in the main text and are not repeated here). As in the left IFG and AG, a 12mm sphere was constructed around the peak voxel of that ROI in each participant’s native space (note that for the right inferior frontal gyrus, the first peak reported was at the edge of the brain, thus we constructed the sphere around another peak in that ROI, MNI coordinates: [50,30,-14]).***

***The data from these additional ROIs is presented in Figure S8. Like in the left IFG analysis, prior to testing asymmetry in representational changes, we examined whether these regions showed any difference in similarity from pre-learning to post-learning. To briefly describe the analysis again here (see main text, Methods and Results for more details), we computed the similarity between multivoxel activity patterns corresponding to the faces that where paired together during the associative learning task. These similarity values are computed before and after learning and a difference score is calculated by subtracting the former from the later. We then average these values for PK pairs and n-PK pairs. We further compare the similarity between paired faces to the similarity between shuffled-pairs, namely faces that did not appear together in the associative learning task. These shuffled pairs serve as baseline. The similarity differences (from pre to post) in each ROI, were submitted to a repeated-measures ANOVA of Prior Knowledge (PK/n-PK) by Pairing (paired/shuffled). In similarity changes from before to after learning, the left middle temporal gyrus (left MTG) and the left orbitofrontal cortex (left OFC) revealed a similar (albeit statistically weaker) pattern to the left IFG , namely, higher similarity for paired faces versus shuffled faces, without an interaction with Prior Knowledge (Pairing main effect: left MTG: F_(1,18)_ = 5.67, p = .029, left OFC: F_(1,18)_ = 4.13, p = .057; main effects of Prior Knowledge and interactions: F_(1,18)_’s < .56, p > .46). The simple effects of paired vs. shuffled faces within each Prior Knowledge pair type did not reach significance (left MTG: PK: t_(1,18)_ = 1.39, p = .18; n-PK: t_(1,18)_ = 1.96, p = .07; left OFC: PK: t_(1,18)_ = 1.64, p = .12; n-PK: t_(1,18)_ = 1.27, p = .22). Of the other ROIs, in the right inferior frontal gyrus (right IFG) we observed a marginal main effect of Prior Knowledge (F_(1,18)_ = 3.19, p = .09; main effect of Pairing, or interaction of Pairing by Prior Knowledge, F_(1,18)_’s < 2.44, p > .13), driven mostly by higher similarity differences for paired n-PK faces (paired vs. shuffled: n-PK: t_(1,18)_ = 2.18, p = .04; PK: t_(1,18)_ = .30, p = .77). The other ROIs (dorsomedial prefrontal cortex, right superior frontal gyrus, left inferior frontal gyrus, dorsal portion) did not exhibit any main effect nor an interaction of Prior Knowledge by Pairing (F_(1,18)_’s < 2.00, p > .17; simple effects of paired vs. shuffled: t_(1,18)_’s < 1.19, p’s > .25).***

***Interestingly, when examining asymmetry in learning, no region has shown the pattern demonstrated by the left IFG. Asymmetry in learning was calculated by computing two similarity values: (1) the similarity of the second face in a pair (B-face) after learning, to the first face in the pair (A-face), and (2) the similarity of the A-face after learning to the B-face before learning, and subtracting the latter from the former. A positive value would mean that the neural representation of the B-face became similar to the A’s face representation during learning (see main text, Methods and Results, for more details). In the main text, we report such positive asymmetry in learning in the left IFG only in PK pairs, suggesting assimilation of new information into prior knowledge structures. As can be seen in Figure S8, no other region showed this pattern. In fact, some regions showed a negative asymmetry value, meaning that the A-face after learning became similar to the B-face before learning (more so than vice-versa). While a couple of regions showed qualitatively negative asymmetry for PK pairs, asymmetry was significantly different for paired compared to shuffled pairs in n-PK pairs in three ROIS (dorsomedial prefrontal cortex, right superior frontal gyrus, and right IFG: t_(18)_’s > 2.2, p’s < .05, in these regions asymmetry for n-PK paired faces was also significantly different from zero t_(18)_’s > 2.50, p’s < .03). This negative asymmetry values might suggest prediction of the B-face upon seeing the A-face (Schapiro, Kustner, & Turk-Browne, 2012). However, note that while in the right IFG asymmetry was found along with a significant increase in similarity from before to after learning in n-PK pairs, the right superior frontal gyrus did not demonstrate such an increase in similarity, and the dorsomedial prefrontal cortex only showed some minor qualitative increase in similarity from before to after learning. Thus, the asymmetry values in these regions should be interpreted with cautious.***

***We now turn to similarity changes from before to after learning specifically for remembered versus forgotten pairs. In the left IFG, we did not observe a significant difference in similarity values based on memory. We did find such differences in the left anterior hippocampus (see Results, main text). The similarity changes from before to after learning in each of the additional ROIs were submitted to repeated-measures ANOVAs with the factors of Prior Knowledge (PK/n-PK) and Memory (Remembered, only high-confidence, as in the main analysis/Forgotten; see main text, Methods and Results). No significant main effects nor interactions were found in any of these ROI (F_(1,17)_’s < 2.56, p > .12).”***

*Figure S8.* Similarity results in cortical ROIs demonstrating functional connectivity with the left anterior hippocampus. Δ Similarity: the similarity difference from before to after learning. Asymmetry: asymmetric changes in similarity. See the text above and main text for more details. For statistical analysis, see the text above.

*Figure SX.* Similarity results in cortical ROIs demonstrating functional connectivity with the left anterior hippocampus. Δ Similarity: the similarity difference from before to after learning. Asymmetry: asymmetric changes in similarity. See the text above and main text for more details. For statistical analysis, see the text above.

It would be useful to see the raw initial and final similarity for each condition in the hippocampus in addition to the change scores, perhaps as a supplemental figure. The interpretation of change scores can vary as a function of initial values.

**Thank you. We note that the values before and after learning might be influenced by autocorrelations among the regressors in the fMRI GLM. Even though we did counterbalance the order of PK and n-PK faces across participants, it is still hard to determine precisely how such autocorrelations tainted the raw similarity values. We additionally had no control over which pairs the participants later remembered or forgot. Therefore, the raw similarity values are difficult to interpret at face value. Critically, the pre-learning scans and the post-learning scans were identical; thus, they contain the same autocorrelation structure. Hence, we can interpret the difference between the initial similarity values (i.e., before learning) and the final similarity values (i.e., after learning; Schapiro et al., 2012). Since the scans are identical, any difference cannot stem from autocorrelations between regressors.**

**Nevertheless, we now report the initial and final similarity values in a supplementary text and figure. As can be seen below, even when looking only at similarity values after learning, the similarity between remembered PK pairs is lower than n-PK pairs. In the Supplementary Information, we added: *“In the main text, we present the difference between similarity before learning and similarity after learning. Here, we present the similarity values from the pre-learning and the post-learning scans separately, for completeness. We note that the similarity values before and after learning are influenced by autocorrelations among the regressors in the fMRI GLM. Even though we counterbalanced the order of PK and n-PK faces across participants, it is still hard to determine precisely how such autocorrelations tainted the raw similarity values. We additionally had no control over which pairs the participants later remembered or forgot. Therefore, due to potential autocorrelations between the activity patterns stemming from the order of the stimuli, the raw similarity values are difficult to interpret at face value. Critically, the pre-learning scans and the post-learning scans were identical; thus, they contain the same autocorrelation structure. Hence, we can only confidently interpret the difference between the similarity values before learning and after learning (Schapiro et al., 2012). As a reminder, when looking at differences from before to after learning, we found that for remembered pairs, the neural representations of faces in PK pairs became less similar to each other through learning, while the representations of faces in the n-PK pairs became more similar (Results, main text).***

***When looking at similarity values in the post-learning scans, the similarity between remembered PK pairs is lower than n-PK pairs (PK: M = .02, SD = .07; n-PK:* *M = .09, SD = .12). We additionally found that in the pre-learning scans, the similarity was higher between remembered PK pairs compared to remembered n-PK pairs (PK: M = .08, SD = .10; n-PK:* *M = .10, SD = .11). Repeated-measures ANOVA, with the factors of Prior Knowledge (PK/n-PK) and Time Point (pre-learning/post-learning) revealed a significant interaction of Prior Knowledge and Time Point (F_(1,17)_ = 8.88, p = .008; simple-effects: pre-learning, PK vs. n-PK: t_(1,17)_ = 1.95, p = .07; post-learning, PK vs. n-PK: t_(1,17)_ = 1.90, p = .07; PK: pre- vs. post-learning: t_(1,17)_ = 3.01, p = .01; n-PK: pre- vs. post-learning: t_(1,17)_ = 2.08, p = .05). The same ANOVA for forgotten pairs revealed no main effect nor an interaction (all F_(1,17)_’s < 1.65, p’s > .21). We emphasize that the current study was not designed to separately examine the pre-learning and post-learning similarity value, as they are likely tainted by autocorrelations between the regressors in the GLM. We therefor refrain from offering a plausible interpretation at this point. Further research using another methodology can carefully examine this pattern of results. Importantly, our results show that similarity differences from before to after learning cannot be attributed to differences before learning alone, as after learning, the similarity between remembered PK pairs was lower than n-PK pairs.”***

**Even though not specifically requested by the reviewer, we reasoned that the asymmetry measure, reported in the left IFG, is a difference score between two values (as detailed below). We now report the similarity values comprising the asymmetry measure in the Supplementary Information. The following text and the figure below were added: *“In the main text we report asymmetry in representational changes in the left inferior frontal gyrus (left IFG). Specifically, we defined asymmetry as the difference between (1) the similarity of the B-face after learning to the A-face before learning, and (2) the similarity of the A-face after learning to the B-face before learning. We found such asymmetry in the left IFG only in the PK pair type, suggesting assimilation of new information into prior knowledge structures (Results, main text). It is informative to examine each of these values separately, to ascertain that indeed the difference stems from positive similarity between the B-face after learning to the A-face before learning, the direction that is consistent with our interpretation (note that each of these similarity values were computed across the pre-learning and post-learning phases, mitigating the concern regarding autocorrelations between regressors in this analysis). As can be seen in Figure S4, indeed the reported asymmetry stemmed from positive similarity between the B-face after learning and the A-face before learning. In PK pairs, this similarity value for paired faces was significantly different from zero (t_(1,18)_ = 3.11, p = .006) or from the same measure calculated for shuffled faces baseline (same faces, but shuffled to compute the similarity such that they are paired with the faces they did not appear with during the associative learning task, see Methods, main text; t_(1,18)_ = 3.15, p = .006). In contrast, the similarity between the A-face after learning and the B-face before learning did not differ from zero or shuffled faces (t_(1,18)_‘s < 1.65, p > .11). The difference between the two similarity measures that are used to compute the asymmetry measure (i.e., the similarity between the B-face after learning and the A-face before learning, and the similarity between the A-face after learning and the B-face before learning) was further significant for paired faces (t_(1,18)_ = 2.71, p = .01), but not for shuffled faces (t_(1,18)_ = 1.69, p = .11). When examining n-PK pairs, none of the above comparisons reached significance (t_(1,18)_‘s < 1.33, p > .20).”***

*Figure S4*. Similarity values comprising the asymmetry measure, left inferior frontal gyrus. “pre”: pre-learning, “post”: post-learning. PK: prior knowledge pairs. N-PK: no prior knowledge pairs. Paired: similarity between faces that were paired during the associative learning task. Shuffled: similarity between the same faces, but the faces are shuffled so that the similarity is computed between faces that did not appear together during the associative learning task. See text for more details and statistical analysis. Error bars reflect +-1 SEM.

I may have missed this, but I’m not sure the authors ran a simple contrast of univariate activity differences between PK and nPK, which would be useful to report.

**We note that this study was not designed to address univariate differences between PK and n-PK pairs. Univariate effects are susceptible to repetition suppression (a reduction in univariate activation due to repetition). We likely had repetition suppression within trials, since we included two rapid presentations of each face in the pair in each trial. Additionally, each double-presentation of the pair repeated in the study 12 times (in 12 cycles), which lead to further suppression across repetitions. Thus, we did not analyze and report univariate differences between PK and n-PK pairs.**

**Nevertheless, to address the reviewer’s request, we now analyzed univariate activation. To that end, we fit a GLM which included a regressor per pair type (12 PK pairs, 12 n-PK pairs) and repetition (we repeated the pairs 12 times during the study, hence, 24 regressors). We then ran a whole brain analysis comparing univariate activity in the first presentation of PK vs. n-PK pairs. In this contrast we sacrificed power in favor of avoiding repetition suppression across trials. This analysis did not reveal any significant cluster for PK > n-PK or the opposite contrast (thresholding of .001, voxel level, and a cluster size of 50 contingent voxels to correct for multiple comparisons at p < .05, determined by Monte-Carlo simulation). In a more lenient threshold (voxel level: p < .005), we find a cluster in the ventromedial prefrontal cortex (vmPFC) demonstrating higher activation for PK compared to n-PK pairs, and a precuneus cluster demonstrating higher activation for n-PK compared to PK pairs (Supplementary Table S2, see below; a cluster of 61 contingent voxels corrects for multiple-comparisons).**

**We additionally examined the comparison between PK and n-PK pairs across all repetitions. This analysis maximizes power, and likely reflects repetition suppression. A few regions including the middle temporal gyrus and the middle frontal gyrus revealed higher activation for n-PK pairs compared to PK pairs, potentially reflecting larger repetition suppression for PK pairs (Supplementary Table S2, see below). No region showed higher activation for PK compared to n-PK pairs, also in the more lenient threshold (as above). These results, as detailed here and including the table below, are now reported in the Supplementary Information.**

|  | *MNI coordinates* | | |  |  |
| --- | --- | --- | --- | --- | --- |
| *Region* | *x* | *y* | *z* | *Z value* | *Num. Voxels* |
| *PK:1^st^ > n-PK:1^st^* |  |  |  |  |  |
| vmPFC | -10 | 48 | -22 | 4.20 | 60 |
|  |  |  |  |  |  |
| *n-PK:1^st^ > PK:1^st^* |  |  |  |  |  |
| left precuneus | -12 | -40 | 44 | 3.78 | 85 |
|  |  |  |  |  |  |
| *n-PK: all > PK: all* |  |  |  |  |  |
| middle temporal gyrus | 44 | -32 | -4 | 4.10 | 97 |
| left middle frontal gyrus | -30 | 40 | 12 | 3.87 | 190 |
| right middle frontal gyrus | 22 | 58 | 28 | 3.65 | 153 |
| left lingual gyrus | -6 | -70 | -2 | 3.64 | 65 |

*Supplementary Table S2*. Univariate activation during the associative learning task. PK: prior knowledge pairs, including famous and novel faces. N-PK: no prior knowledge pairs, including two novel faces. 1^st^: only first presentation of the pairs. All: all repetition of the pairs.

I puzzled over the claim in a couple places that RSA but not univariate/connectivity methods provides information about “content”. Maybe this claim could be unpacked. RSA does provide information that is specific to individual items, but what endows this method in particular with the ability to see “content”? Similarly, in the Discussion, the authors claim that this study, unlike prior ones, can address what “learning process” is taking place. I’m not sure about this as a qualitative distinction. This study shows representational changes that could be a result of many different kinds of learning processes. Perhaps RSA is a more constraining kind of measurement, but I think the claim that it provides special access to content and learning mechanisms would need some defending.

**Thank you for this comment. We have tried to refer to the notion of a neural representation in simpler terms. The main point we wished to convey is that univariate findings cannot inform about processes such as separation or assimilation, whereas RSA can at least gauge these processes, at the level of voxels. We are in agreement with the reviewer and acknowledge that our wording might have led to a misunderstanding of our interpretation. We have modified the manuscript and removed references to content or learning processes in several places:**

1. **A sentence in the abstract is now modified: *“Remarkably, however, how prior knowledge influences the neural representation of novel associations remains unknown.”*** **(p. 2).**
2. **In the introduction, we shortened a sentence by removing “content of learning” from the sentence: *“Critically, however, univariate activation and functional connectivity studies cannot address how prior knowledge modulates the neural representation of novel associations in these memory systems”.*** **(p. 4).**
3. **In the first paragraph of the Discussion, we modified the sentence the reviewer referred to. The new sentence is: *“However, as previous studies only looked at the strength of brain activation and lesions, they could not address the neural representations of new associations. Thus, the question of whether prior knowledge promotes separation versus assimilation in different neural systems remained unexplored to date.”*** **(p. 16).**
4. **In a section in the discussion that describes cortical learning, we modified a sentence: *“Critically, while previous empirical findings of cortical activation and functional connectivity lend some support to the cortical assimilation idea (Liu et al., 2016; Maril et al., 2011; Reggev et al., 2016; Staresina et al., 2009; van Kesteren, Rijpkema, et al., 2010), they could not address whether and how assimilation occurs at the level of the neural representation.”* (p. 21).**

Minor: In the supplement, line 65, there is a sentence that ends prematurely.

**Thank you. This section is now in the main text, so this sentence was removed from the supplement, and was modified in the main text (see above).**

Reviewer #2 (Remarks to the Author):

Summary of Paper

This paper had two main hypotheses: (1) when a novel item is being associated to a familiar item (existing knowledge), the cortical system is biased towards assimilation whereas the hippocampus is biased towards pattern separation to prevent interference; (2) learning this association results in asymmetric cortical effects in that novel items, compared to familiar items, will go through relatively larger transformations. Using fMRI, the researchers found evidence that supported their hypotheses: (1) multivoxel activity patterns representing familiar-novel pairs became separated in the hippocampus whereas the novel item in the novel-familiar pair became more similar (i.e. integrated) to the familiar item after learning, and (2) positive asymmetry was found for famous-novel pairs but not for novel-novel pairs. Altogether, these findings support the influence of existing knowledge on learning.

Summary of Comments

The direction of assimilation and the observed asymmetric cortical effects are exciting findings. While the study contributes important additions to the literature, the paper requires substantial edits to enhance clarity of methodology and logic in the analyses conducted.

**We thank the reviewer for the positive evaluation of our work.**

Major Comments

For ease of commenting, I refer to:
● F1 as the pre-learning famous face
● F2 as the post-learning famous face
● N1 as the pre-learning novel face that is eventually paired with the famous face
● N2 as the post-learning novel face that was paired with the famous face
● r is representational similarity

1. While the current study computed r(F2,N2) - r(F1,N1) to provide evidence for pattern separation, one also can assess pattern separation in another way: assuming that F2 changes with learning, one could compare 1) the change in representation of the famous face in the hippocampus (F2 in HPC - F1 in HPC) to 2) the change in representation of the famous face in the cortex (F2 in cortex - F1 in cortex). If pattern separation is occurring in HPC and assimilation in the cortex, 1) would be larger than 2). It may be worthwhile to conduct this analysis to support your current evidence.

**We appreciate this thoughtful suggestion and have now conducted this analysis as detailed in the reviewer’s comment. We took only remembered pairs, as these are the pairs that showed separation in the left anterior hippocampus. We found that the similarity (F2-F1) was indeed lower in the left anterior hippocampus than in the cortex (left anterior left inferior frontal gyrus, left IFG: *M* = .017, *SD* = .05; Hippocampus: *M* = .003, *SD* = .09). However, as can be seen in the SDs, there was large variance in the data, and the qualitative difference did not reach statistical significance (*t_(17)_* = .62, *p* = .52). This is an interesting additional analysis, and the results are qualitatively as expected. However, since the results were not statistically significant, we decided not to report them in the paper. If the reviewer believes it is useful, we are happy to include this analysis in the supplementary and provide some speculation as to why we believe this measure might be noisier than the other measures used in the manuscript.**

2. When assessing whether N2 has become integrated with the existing representation of the famous face, the authors assume that this integration occurs between F1 and N2, rather than F2 and N2. (i.e. to assess asymmetric cortical updating they calculate the similarity between the representation of a novel face after learning to the original representation of the famous face with which it was associated.) Why is it assumed that the novel face becomes assimilated into the original face representation, rather than the updated face representation (F2)? In real-time integration, the concept space may become modified with the addition of a new item. Integration of the novel face in the existing face representation may co-occur with the updating of the existing face representation, thus resulting in N2 becoming integrated with F2 rather than F1.

**We thank the reviewer for this comment as it shows that, regrettably, we were not clear enough in explaining our analyses and presenting the data in the previous version of the manuscript. We did examine the similarity between F2 and N2 and found that similarity *increased* from pre-learning to post-learning. Further, this increase was significantly different from shuffled pairs that were used baseline** (**paired vs. shuffled: *t*_(18)_ = 4.1, *p* = .0007, Cohen’s *d* = .94; p. 12; Shuffled pairs were made by pairing each famous face with novel faces that appeared with other famous faces. This controls for changes in similarity that are not specific to the pairing of faces together, see Methods). Thus, we did observe integration in the way that the reviewer is suggesting. We previously included this result in the Supplementary Information, but to increase clarity and visibility and also following Reviewer #1’s comment, this result is now reported in the main text. The following section was added, prior to describing the asymmetry results: “*Although we targeted asymmetric changes in the representational patterns from before to after learning, we first examined whether any changes in representational similarity occurred from before to after learning. To that end, we asked whether there was evidence of learning-related similarity changes from pre- to post-learning in the left IFG. For each pair type (PK/n-PK), we compared the change in representational similarity between pairs of faces that appeared together during the learning task to the shuffled pairs baseline (faces from the same PK/n-PK pair-type that did not appear together at learning, see Methods). This comparison allowed us to specifically examine changes due to associative learning, controlling for similarity differences due to item familiarity or type of pair (Schapiro et al. 2012). A repeated-measures ANOVA with Prior Knowledge (PK/n-PK) and Pairing (paired/shuffled), revealing a main effect of Pairing (F_(1,18)_ = 11.44, p = .003, η_p_^2^ = .32), and no main effect of Prior Knowledge or Prior Knowledge by Pairing interaction (F’s < 1.2, p’s > .29). Pairwise comparisons showed a highly significant increase in similarity in PK pairs (PK: paired: M = .03, SD = .04; shuffled: M = -.01, SD = .02; t_(18)_ = 4.1, p = .0007, Cohen’s d = .94). N-PK paired faces also became qualitatively more similar compared to shuffled baseline (paired: M = .02, SD = .06; shuffled: M = .00, SD = .02; t_(18)_ = 1.16, p = .26). Thus, we obtained greater increase in similarity specifically for paired faces, but not for shuffled faces, indicating that there was a change in similarity from before to after learning.“* (p. 12).**

**As explained in the Results section, however, this measure of similarity only tells us that the representations of the faces became more similar, or more integrated, but it cannot inform us about the direction of the integration. When computing the similarity as above (namely, between F2 and N2), we have one correlation value between the multivoxel activity patterns corresponding to the faces after learning (which tells us how similar their representations were after learning), and we subtract from that the correlation before learning (the similarity between F1 and N1) as baseline. While this tells us that the faces became similar, we cannot infer based on this measure whether, during learning, that novel face became similar to the famous face, or vice-versa. Computing the similarity between N2 and F1 allows us to examine how similar is the novel face after learning, to the representation of the famous face before learning, as a way of gauging whether during learning, the representation of the novel face became similar to that of the famous face. From that value, we can subtract the similarity between F2 and N1, and that leads to the asymmetry index we used, that indicates whether the neural representation of the novel face became more similar to that of the famous face during learning than vice-versa. While we definitely agree that N2 should be similar to F2, and indeed our data are consistent with that, the use of the similarity between the representations of N2 and F1 (and likewise, F2 and N1) was done to enable looking at the direction of similarity. This was explained in the previous version of the manuscript, but might have not been clear enough. To improve clarity, we now incorporate the F2-N2 similarity in the main text, as detailed above. We also modified the explanation of the asymmetry analysis: “*When comparing the representational similarity of items after learning, we only get one measure of the magnitude of similarity between the neural representations of faces in the pairs. It is thus unclear whether similarity increase reflects changes in the representations of both items, such that they both become more similar to each other (symmetric changes), or in one item, which becomes more similar to the other (asymmetric changes). To test our main hypothesis regarding asymmetry in learning, we reasoned that comparing the post-learning pattern of the novel B-face to the pre-learning pattern of its paired famous A-face would give us a pure measure of how much the representation of novel B-face became more similar to the representation of the famous A-face during learning (we performed an identical procedure for novel-novel n-PK pairs). Thus, we compared the representational similarity between the B-face after learning (always novel) and the A-face (famous or novel) before learning, as well as the representational similarity between the A-face after learning and that of the B-face before learning. We then subtracted the latter from the former to get an asymmetry measure: if both face representations became equally more similar to one another, there would be no difference between these two values. If, however, the B-face after learning became more similar to the A-face before learning, while the A-face did not become more similar to the B-face before learning, then we should observe a positive value, indicating asymmetry (Schapiro et al., 2012; Methods and Figure 4).”* (p. 13).**

**We hope that these changes make the asymmetry analysis clearer.**

Minor Comments

1. Line 111 – The idea that existing representations are stronger is one that hasn’t been introduced beforehand; this assumes the reader knows what makes a representation “stronger” than another.

**We acknowledge the confusion. In a second read, we thought that the word “stronger” might be redundant altogether, so we removed it from the sentence: *“To illustrate, since a cortical representation of Madonna has been stabilized over a lifetime of exposure, it might only change slightly to incorporate the novel association with her friend. The representation of the friend, however, will undergo a disproportionately larger transformation during learning, in order to be assimilated into our existing representation of Madonna.”* (p. 5).**

2. Line 180, line 301 – the expectation for increased similarity in hippocampus for novel items was not set up in the Introduction and comes across as an unexpected finding.

**Thank you. We expected increased similarity in the hippocampus for novel items based on a prior study (Schapiro et al., 2012). In the interest of a streamlined Introduction, we opted to mention this study in the beginning of the section dedicated to the hippocampal findings: *“We tested whether the hippocampal representations of two associated items become more distinct when learning involved prior knowledge. This is in contrast to novel items that do not involve prior knowledge. In this case, a prior study using novel visual fractals has shown that after learning, the hippocampal representations of paired fractals became more similar to each other (Schapiro et al., 2012).“* (p. 7). We now also mention that our similarity results is consistent with the prior study in the Discussion (previously line 301): “*We note that the result in the novel-novel face pairs is consistent with a previous study that found increased similarity in the hippocampus after learning of pairs of novel visual fractals (Schapiro et al., 2012). Critically, this previous study did not address prior knowledge, nor were the previous findings related to subsequent memory in that study.”* (p. 16).**

**We thank the reviewer, as we believe that emphasizing the reliance on that prior study and the consistency in the findings further strengthens our results. We could also incorporate this finding into the Introduction, if the reviewer finds it preferable.**

3. Line 838 – beyond pixel correlations, were other details controlled for such as emotional valence or hair length?

**Yes. As was mentioned in the methods, we did control other factors such as age. We also used photos of foreign corporate executives, actors, and models that were unfamiliar to our Israeli participants, to control for such as attractiveness and image quality (p. 34). Hair length was not explicitly controlled for, as we expected hair length and other visual properties to reflect in the pixelwise similarity. As per emotional valance, all of our faces had neutral to moderately-positive expressions. In addition to these controls, we note that we had a stringent within pair-type control, namely, the shuffled pairs. Specifically, shuffled pairs are composed of the same faces that are used for the main analysis, they have appeared in the experiment in the same PK/n-PK pair type, but critically, faces were shuffled such that the representational similarity was computed between pairs of faces that did not appear in the study together. Since the shuffled faces are the same faces as the paired faces, they have the same visual and other properties. The only difference for calculating the similarity values for paired vs. shuffled pairs is in how faces were paired to compute the correlation. Any influence of face-specific properties, or other factors that are not due to faces being paired together in the study, should have been expressed in the shuffled faces as well. Nevertheless, the results we report in the manuscript were specific to faces that were paired together during the associative learning task. We therefore find it unlikely that visual properties or valance can account for our findings.**

4. Lines 841-842 require rewording to clarify the methodology. Perhaps replace with “2 B-faces were paired with each A-face pair. These pairings were random for each participant, although they were fixed within the experiment…”

**We apologize for the potential misunderstanding. We note that in the experiment, each pair had one A-face and one B-face. For each participant, each A-face was only paired with one B-face throughout the experiment (in all 12 repetitions of the pair). We randomized the pairing between A and B faces across all 24 A-faces and 24 B-faces. We have now modified the text in the manuscript: *“The experiment included pairs of face that were presented together, each pair comprised of one A-face (either famous or novel, see below) and one B-face (always novel). The pairing of the each of the 24 novel B-faces with each of the 24 A-faces (12 famous, 12 novel) was random for each participant. Within participant, the pairings were fixed throughout the experiment (i.e., for each participant, each A-face appeared with one B-face, and the same pair appeared in all the repetitions, see below)”* (p. 34).**

**We hope that the current explanation is clearer.**

5. Line 874 – What was the purpose of this “irrelevant task”?

**The irrelevant task was meant to be used in future studies in the lab. It comprised a “localizer” scan in which participants were presented with famous faces, novel faces and objects (different famous and novel faces than the ones used in the main experiment). Previous research has identified brain regions involved in processing of famous faces versus novel faces (Gobbini & Haxby, 2007; Natu & O’Toole, 2011). Due to the lab’s interest in prior knowledge effects, in future studies in the lab, we wish to examine whether we can use this type of a task to identify regions involved in processing of famous faces compared to novel faces within each single participant. Since we had participants in the scanner, we asked them to perform this additional task.**

6. Lines 891-895 require clarification because there is a jump in the logic. What do you mean by “regressor of that face”? This concept was inadequately introduced/explained.

**Thank you, this was an unnecessary jump. This sentence is now modified:** ***“To create the pseudo-randomized order, placeholders of stimuli were fixed, e.g., a certain face appeared in triad of placeholders at locations 20, 150, and 180. Placeholders triads were then paired such that two faces that would be associated later would each appear in one placeholder triad of the pair (e.g., placeholders 20, 150, and 180 were paired with placeholders 40, 105, and 240).”* (p. 36).**

7. Line 940 – aren’t the three faces that appear at the bottom of the screen 2 B-faces and 1 A-face (the other item in the A-face pair?) If all novel faces are supposed to be B-faces (even the one that was paired to the famous face), then lines 841-842 need to be revised.

**We hope that our modifications above have clarified that during the study, each A-face was only paired with one B-face. In the associative memory test, an A-face appeared at the top of the screen, and participants had to indicate which of the 3 faces at the bottom of the screen appeared with that A-face. The three faces at the bottom of the where thus the target B-face that appeared with the A-face on the top during learning, and 2 additional B-faces that appeared with other A-faces of the same pair type (PK/n-PK).**

Line-by-Line Edits

**Thank you for the useful edits. We have incorporated them in the current version of the manuscript. Below, we only respond where we did not follow the exact edit suggested by the reviewer.**

1. Line 24 – remove “together”
2. Line 28 – remove the hyphen between “highly” and “familiar”
3. Line 44 – switch the order of “media posts” and “ads” to parallel logic of “…new album or starts dating…”
4. Line 45 – move towards active voice rather than passive. E.g. “How do we form such new associations?”
5. Line 63 – after “lovers” change n-dash to m-dash
6. Line 130 – “post-learning” instead of “post-similarity”
7. Line 277 – “how individual associations are…” to parallel previous structure
8. Line 286 – remove “critically” -- too repetitive.

**Thanks. This sentence now opens with “However”.**
9. Line 308 – colon instead of comma

**We removed the comma and the word that followed it, to allow a better flow of the sentence: *“Together, these findings show a flexible and directional creation of associations in the human brain that is specific to memory systems and is highly modulated by the state of prior knowledge.”* (p. 17).**
10. Line 388 – remove “asking” and rephrase sentence

**The current sentence is: *“Researchers have investigated the processes as well as the timeline that characterizes cortical learning for decades”.* (p. 21).**
11. Line 815 – add hyphen in “two-thirds”
12. Line 848 – choose “by”, “x” or “*” for describing resolution and keep consistent
13. Lines 872-873 – restructure sentence to move emphasis off of testing and onto the post-learning scan. E.g. “Critically, acquiring the post-learning scans before testing memory allowed us to measure post-learning representations without interference…”
14. Line 973 – add “e” to Siemens
15. Line 978 – add “/” instead of hyphen in pre/post


Figure Edits

1. Figure 1.d) – “confidence”

**Thank you. This is now corrected.**

Reviewer #3 (Remarks to the Author):

Bein and colleagues report and fMRI study in which they manipulated the extent to which new associations could be incorporated into prior knowledge. They accomplished this by having half of the pairs include one famous (e.g., Madonna) and one novel face (PK pairs); the other pairs were two novel faces (n-PK pairs). They found that the representation of the two paired faces changed from pre- to post- learning in a way that was dependent on the prior knowledge manipulation: in anterior hippocampus, items in PK pairs became less similar with learning, while n-PK pairs became more similar. The same anterior hippocampal region showed greater connectivity with a network of neocortical regions (including LIFG) specifically during PK encoding. LIFG also showed an increase for the PK pairs which was driven by the novel face coming to be represented more similarly to the famous face ("assimilation").

I am generally enthusiastic about the topic and this paper. The manuscript itself is generally clear and well-written, and the results are very interesting.

**Thank you very much. We appreciate the enthusiasm and the positive evaluation of our work.**

I do have some concerns and questions with respect to interpretation and methods, however.

Major comments:

1. To what extent are the findings about asymmetry specific to LIFG? First of all, the section is titled "cortex," which is extremely vague and borderline misleading, as the authors only report one of their (several) cortical regions. I understand the idea is to align this with CLS but the authors are not showing assimilation in "cortex," but rather just IFG. Please retitle this to be about LIFG if that is the only region that will be discussed. I think the paper title should be revised for the same reason. Second, why were the other functional regions (derived from the same analysis as the IFG region that was interrogated; shown in Figure 3) not tested? Perhaps it is specific to IFG (which would be interesting), but perhaps it is not (which in my mind is also interesting). Either way, I think it's important to know the answer to this.

**We thank the reviewer for this comment. We modified the title of section addressing the left IFG results to: “*Left inferior frontal gyrus: New information is assimilated into cortical knowledge structures”* (p. 11).**

**We additionally modified the abstract. Instead of referring to “cortex”, we now specifically indicate the left inferior frontal gyrus: *“In the left inferior frontal gyrus, however, prior knowledge led to integration, and in a specific direction: the representation of the novel face became similar to that of the famous face after learning, suggesting assimilation of new into old memories.”* (p. 2).**

**As for the title of the manuscript, it can certainly be changed to “Hippocampal separation and cortical assimilation: prior knowledge promotes divergent codes in the hippocampus versus the left inferior frontal gyrus”. While we acknowledge the reviewer’s concern, we found that this made the title less elegant than our previous title, and that the other changes are useful to prevent misunderstanding of the results. However, if the reviewer prefers, we can modify the title as well, or leave it to editorial considerations.**

**Regarding our focus on the left IFG and additional cortical regions, as is mentioned in the manuscript (p. 11), we focused on the LIFG due to its known involvement in prior knowledge influences on new learning (Maril et al., 2011; Reggev et al., 2016; Staresina et al., 2009), as well as more broadly in semantics (Badre, Poldrack, Paré-Blagoev, Insler, & Wagner, 2005; Badre & Wagner, 2007; Binder & Desai, 2011). As we noted in a foot note in the previous version of the manuscript, we have additionally looked at the Angular Gyrus (AG), since it was recently proposed to bind aspects of schematic knowledge and mediating schema influences on encoding (Bein, Reggev, & Tompary, 2018; Gilboa & Marlatte, 2017; van der Linden, Berkers, Morris, & Fernández, 2017; I. C. Wagner et al., 2015). In the AG, we found no significant similarity changes from pre- to post-learning, with or without respect to subsequent memory (ANOVA’s of Prior Knowledge by Paired/Shuffled, or of Prior Knowledge by Memory, F’s < 2.08, p’s > .16). We thank the reviewer for pointing out that this was not clear enough in our previous version of the manuscript. We now added the results from the AG to the main text: “*We further examined whether asymmetry in learning was observed in other cortical regions that demonstrated functional connectivity with the left anterior hippocampus. We chose the angular gyrus (AG), since it was recently proposed to bind aspects of schematic knowledge and mediate schema influences on encoding (Bein, Reggev, & Tompary, 2018; Gilboa & Marlatte, 2017; van der Linden, Berkers, Morris, & Fernández, 2017; I. C. Wagner et al., 2015). In the AG, we found no similarity differences from pre- to post-learning, with or without respect to subsequent memory (ANOVA’s of Prior Knowledge by Paired/Shuffled, or of Prior Knowledge by Memory, F’s < 2.08, p’s > .16). For completeness, we have directly targeted asymmetry, which did not differ from zero or shuffled pairs in either PK or n-PK pairs (all t_(18)_’s < 1.46, p’s > .16).”* (p. 13).**

**Following this reviewer and Reviewer #1’s recommendation, as an exploratory analysis, we have now additionally examined additional cortical regions showing connectivity with the hippocampus. We report these results in full in the Supplementary Information and refer to them in the main text. In the main text, we added: *“As an exploratory analysis, we have examined the other cortical regions that demonstrated functional connectivity with the left anterior hippocampus. The data from these regions are reported in the Supplementary Information. Interestingly, even though some of the regions showed an overall increase in similarity from before to after learning, no other region that we examined has shown asymmetry in the direction of representational changes, as was observed in the left IFG. “* (p. 14).**

**In the Supplementary Information, we now added the following text and figure:**

***“In the main text, we report a number of regions that demonstrated higher functional connectivity for PK pairs compared to n-PK pairs (main text, and above, Table S1). Of these, we focused on the left inferior frontal gyrus (left IFG) and the angular gyrus (AG), due to prior literature (see main text). As an exploratory analysis, we examined whether the additional cortical regions that demonstrated functional connectivity with the hippocampus showed asymmetry in the representational changes, as was observed in the left IFG (the left IFG and the AG are reported in the main text and are not repeated here). As in the left IFG and AG, a 12mm sphere was constructed around the peak voxel of that ROI in each participant’s native space (note that for the right inferior frontal gyrus, the first peak reported was at the edge of the brain, thus we constructed the sphere around another peak in that ROI, MNI coordinates: [50,30,-14]).***

***The data from these additional ROIs is presented in Figure S8. Like in the left IFG analysis, prior to testing asymmetry in representational changes, we examined whether these regions showed any difference in similarity from pre-learning to post-learning. To briefly describe the analysis again here (see main text, Methods and Results for more details), we computed the similarity between multivoxel activity patterns corresponding to the faces that where paired together during the associative learning task. These similarity values are computed before and after learning and a difference score is calculated by subtracting the former from the later. We then average these values for PK pairs and n-PK pairs. We further compare the similarity between paired faces to the similarity between shuffled-pairs, namely faces that did not appear together in the associative learning task. These shuffled pairs serve as baseline. The similarity differences (from pre to post) in each ROI, were submitted to a repeated-measures ANOVA of Prior Knowledge (PK/n-PK) by Pairing (paired/shuffled). In similarity changes from before to after learning, the left middle temporal gyrus (left MTG) and the left orbitofrontal cortex (left OFC) revealed a similar (albeit statistically weaker) pattern to the left IFG , namely, higher similarity for paired faces versus shuffled faces, without an interaction with Prior Knowledge (Pairing main effect: left MTG: F_(1,18)_ = 5.67, p = .029, left OFC: F_(1,18)_ = 4.13, p = .057; main effects of Prior Knowledge and interactions: F_(1,18)_’s < .56, p > .46). The simple effects of paired vs. shuffled faces within each Prior Knowledge pair type did not reach significance (left MTG: PK: t_(1,18)_ = 1.39, p = .18; n-PK: t_(1,18)_ = 1.96, p = .07; left OFC: PK: t_(1,18)_ = 1.64, p = .12; n-PK: t_(1,18)_ = 1.27, p = .22). Of the other ROIs, in the right inferior frontal gyrus (right IFG) we observed a marginal main effect of Prior Knowledge (F_(1,18)_ = 3.19, p = .09; main effect of Pairing, or interaction of Pairing by Prior Knowledge, F_(1,18)_’s < 2.44, p > .13), driven mostly by higher similarity differences for paired n-PK faces (paired vs. shuffled: n-PK: t_(1,18)_ = 2.18, p = .04; PK: t_(1,18)_ = .30, p = .77). The other ROIs (dorsomedial prefrontal cortex, right superior frontal gyrus, left inferior frontal gyrus, dorsal portion) did not exhibit any main effect nor an interaction of Prior Knowledge by Pairing (F_(1,18)_’s < 2.00, p > .17; simple effects of paired vs. shuffled: t_(1,18)_’s < 1.19, p’s > .25).***

***Interestingly, when examining asymmetry in learning, no region has shown the pattern demonstrated by the left IFG. Asymmetry in learning was calculated by computing two similarity values: (1) the similarity of the second face in a pair (B-face) after learning, to the first face in the pair (A-face), and (2) the similarity of the A-face after learning to the B-face before learning, and subtracting the latter from the former. A positive value would mean that the neural representation of the B-face became similar to the A’s face representation during learning (see main text, Methods and Results, for more details). In the main text, we report such positive asymmetry in learning in the left IFG only in PK pairs, suggesting assimilation of new information into prior knowledge structures. As can be seen in Figure S8, no other region showed this pattern. In fact, some regions showed a negative asymmetry value, meaning that the A-face after learning became similar to the B-face before learning (more so than vice-versa). While a couple of regions showed qualitatively negative asymmetry for PK pairs, asymmetry was significantly different for paired compared to shuffled pairs in n-PK pairs in three ROIS (dorsomedial prefrontal cortex, right superior frontal gyrus, and right IFG: t_(18)_’s > 2.2, p’s < .05, in these regions asymmetry for n-PK paired faces was also significantly different from zero t_(18)_’s > 2.50, p’s < .03). This negative asymmetry values might suggest prediction of the B-face upon seeing the A-face (Schapiro, Kustner, & Turk-Browne, 2012). However, note that while in the right IFG asymmetry was found along with a significant increase in similarity from before to after learning in n-PK pairs, the right superior frontal gyrus did not demonstrate such an increase in similarity, and the dorsomedial prefrontal cortex only showed some minor qualitative increase in similarity from before to after learning. Thus, the asymmetry values in these regions should be interpreted with cautious.***

***We now turn to similarity changes from before to after learning specifically for remembered versus forgotten pairs. In the left IFG, we did not observe a significant difference in similarity values based on memory. We did find such differences in the left anterior hippocampus (see Results, main text). The similarity changes from before to after learning in each of the additional ROIs were submitted to repeated-measures ANOVAs with the factors of Prior Knowledge (PK/n-PK) and Memory (Remembered, only high-confidence, as in the main analysis/Forgotten; see main text, Methods and Results). No significant main effects nor interactions were found in any of these ROI (F_(1,17)_’s < 2.56, p > .12).”***

*Figure S8.* Similarity results in cortical ROIs demonstrating functional connectivity with the left anterior hippocampus. Δ Similarity: the similarity difference from before to after learning. Asymmetry: asymmetric changes in similarity. See the text above and main text for more details. For statistical analysis, see the text above.

*Figure SX.* Similarity results in cortical ROIs demonstrating functional connectivity with the left anterior hippocampus. Δ Similarity: the similarity difference from before to after learning. Asymmetry: asymmetric changes in similarity. See the text above and main text for more details. For statistical analysis, see the text above.

2. I kept thinking to myself that the involvement of LIFG in language seems to be important here, since the famous faces critically differ from the non-famous faces in how nameable they are. Perhaps participants are bringing to mind the verbal label ("Madonna") and this is driving the apparent asymmetry. Do the authors suspect this is what is going on? To what extent is this an issue for the interpretation, if at all? I think the authors intend for the "Madonna" schema to be more than the verbal label itself. This feels like an important limitation of and discussion point for the study, and one that is especially true in the realm of face stimuli. (For example, both famous and non-famous locations/scenes are somewhat nameable, though albeit to different degrees or levels of specificity.)

**We thank the reviewer for this insightful comment and interpretation. While this is definitely a possibility, we find it less likely. First, if the participants are bringing to mind the verbal label of “Madonna” when they see the B-face that was attached to Madonna, that should reflect not only on our asymmetry measure, but also simply on the changes in similarity from pre-learning to post-learning. However, in overall changes from pre-learning to post-learning, the similarity differences between PK and n-PK (where the A-face was novel, and thus had no verbal label) were far from being significant (p > .43). Second, if indeed participants recalled Madonna’s name upon seeing the B-face, we would expect to see asymmetry in other regions as well. For example, in the left angular gyrus, or the hippocampus - brain regions known to be involved in recollection (e.g., Hayama, Vilberg, & Rugg, 2012; Rugg & Vilberg, 2013; Thakral, Wang, & Rugg, 2017). However, these regions did not exhibit asymmetry (for the asymmetry results in the hippocampus, see below), nor did the other cortical regions we examined. Although we find this interpretation less viable, we now explicitly address this possibility together with point #3 below in the Discussion. As note below, we further think that even if true, this possibility might contribute to assimilation: “*Another possibility is that the novel B-face brings to mind the famous A-face because it is well-known and namable, while the novel B-face is not nameable and harder to bring to mind upon seeing the A-face. For example, it may be that during the post-learning scans, when seeing the B-face that was paired with Madonna, the participants thought “Madonna”, but in contrast, the novel and unnamable B-face was not recalled upon seeing Madonna. In our view, this possibility is less likely. First, if the participants were bringing to mind Madonna when they saw the B-face that was paired with Madonna, that should have reflected on the overall similarity changes from pre-learning to post-learning. However, differences in similarity changes from pre-learning to post-learning between PK and n-PK were far from being significant in the left IFG. Second, if indeed participants recalled Madonna’s name upon seeing the B-face, we would expect to see asymmetry in other regions as well. For example, in the left angular gyrus, or the hippocampus - brain regions known to be involved in recollection (e.g.,*** (Hayama et al., 2012; Rugg & Vilberg, 2013; Thakral et al., 2017)***). However, these regions did not exhibit asymmetry, nor did the other cortical regions we examined (see Supplementary Information). Thus, we believe that this interpretation is less likely than the assimilation interpretation. Further, even if indeed the B-face brings to mind the famous A-face, it might be that this process facilitate assimilation, as it renders the B-face’s representation similar to the famous A-face. Future research could better elucidate the processes by which assimilation takes place.”*** **(p. 23).**

3. I do not share the authors' intuition that asymmetry going in the B-->A direction cannot mean that B is not bringing to mind A (p. 19). It is true that it is in the opposite direction of the temporal relationship, but that is only one dimension that matters. (Also as I understand it, since each pair was repeated twice ABAB with no gap, that means that B does come before A?) Surely direction/ease of movement within semantic or associative networks need also be considered. Here, the A item is the (by design) more well-known stimulus; why would it be surprising that B would trigger retrieval of A? Certainly the ease and fidelity of reactivation of a well-known (famous) face would be expected to be higher (B-->A) than a novel face (A-->B). This is somewhat related to my point about the verbal labels (famous people's names).

**For the reasons mentioned above, we find this interpretation less likely. We are thankful to the reviewer for raising this possibility, and we now address this interpretation in the Discussion (see above).**

4. Was there a relationship between the amount of dissimilarity in hippocampus and the amount of similarity in LIFG? Ideally the authors could ask this on a trial-wise basis to determine whether hippocampal separation and LIFG assimilation are occurring together on the same set of trials (i.e., are positively related), or across different trials (unrelated or perhaps even negatively related). Knowing the nature of this relationship might change or strengthen the authors' interpretation.

**We appreciate the thoughtful suggestion and have now ran this analysis and found some supportive evidence for a correlation between asymmetry in the left IFG and separation in the hippocampus. We used a linear mixed-level model approach where trial-by-trial asymmetry scores in the left IFG were the explained variable, and similarity differences from pre-learning to post-learning in the hippocampus were the explaining variable. We looked at PK pairs, in which assimilation occurred, and we broke this analysis to remembered (high-confidence responses only, as in the main analysis) and forgotten pairs, as hippocampal separation was only observed for remembered pairs. Thus, if any relationship exists between hippocampal separation and left IFG assimilation, we should expect to find it in remembered pairs. Statistical significance was determined by comparing the model including the factor of interest to an identical model excluding the factor of interest. All models included an intercept per participant. First, we examined only remembered pairs and found that similarity changes in the hippocampus significantly explained variance in asymmetry in the left IFG (*χ*^2^** **= 4.15, *p* = .04, AIC reduction: 2, equivalent BIC scores: difference of +.19; see figure below). We additionally ran two control analyses. First, we examined specificity to remembered trials, by including both remembered and forgotten pairs and testing the interaction of memory (remembered vs. forgotten) with hippocampal similarity. This interaction was marginally significant (*χ*^2^ = 3.41, *p* = .06, AIC reduction: 1.5, but BIC increase: 1.9). Second, we examined whether the observed correlation was specific to pairs that appeared together during the associative learning task. For both for the left IFG and the hippocampus, we took for each pair, the asymmetry (or similarity difference in the hippocampus) computed between the A-face and its paired B-face, and subtracted from it the same measure, but computed between that A-face and all other B-faces that appeared in with other PK A-faces (i.e., we subtracted the asymmetry/similarity differences for shuffled pairs). Taking this measure that is a pair-specific measure, we again obtained a marginal correlation (*χ*^2^ = 3.37, *p* = .06, AIC reduction: 1.5, but BIC increase: 1). These results provide some preliminary evidence that indeed pairs with higher hippocampal separation, also exhibited larger asymmetry in the left IFG. However, we note that the correlation is moderate, and the control analyses did not reach significance. Another study, potentially with more statistical power, might better elucidate this relationship.**

**These results, albeit suggesting an interesting correlation, did not seem strong enough to be included in the main text. We therefore opted to report the results as described here and including the figure below, in the Supplementary Information. In the main text, after reporting the results in the left IFG, we mention: *“Interestingly, we also find some preliminary evidence that asymmetry in the left IFG might be correlated with hippocampal separation (see Supplementary Information).”* (p. 13).**

*Figure S2*. Hippocampal separation correlated with left inferior frontal gyrus (left IFG) assimilation. PK: prior knowledge pairs, including a famous face and a novel face. ΔSimilarity: similarity post-learning minus similarity pre-learning. Asymmetry: asymmetric changes in similarity. Data is from PK pairs only. See text for details.

5. Was there asymmetry in the movement of hippocampal representations? Or, why wasn't this investigated?

**We had no hypothesis for asymmetry in the changes in hippocampal representations, so we did not examine asymmetry in the hippocampus previously. We have now run this analysis, namely, we computed the same asymmetry measure as was done for the left IFG and is detailed above (and in the Methods and Results in the main text). As a reminder, in the left anterior hippocampus, we found similarity difference from before to after learning specifically for remembered pairs, but not for forgotten pairs. Therefore, we averaged the asymmetry values separately for remembered and forgotten pairs. We found no evidence for asymmetry in learning in the left anterior hippocampus. The asymmetry values for PK or n-PK pairs, either remembered or forgotten, did not differ from zero or from each other (Remembered: PK: *M* = .004 , SD = .10, n-PK: *M* = .025 , SD = .15; Forgotten: PK: *M* = .008 , SD = .11, n-PK: *M* = .028 , SD = .17; all *t’*_(17)_ < 1.64, *p*’s > .11). We now report these results in the Supplementary Information.**

6. Since the analysis was performed for only those trials that were remembered, it would be important to demonstrate that the observed representational change differences are not due to differences in the number of trials and thus the stability of estimates between the conditions. This could be accomplished by subsampling the condition with the larger number of comparisons and re-performing the analyses.

**We thank you for this useful comment. We have run the suggested analysis and the results were consistent. Specifically, for each participant, we identified the condition with the least number of trials across the 4 conditions (PK/n-PK by remembered/forgotten), and randomly selected the same number of trials in the other conditions. Then, we computed the average pre-post similarity difference across the subsampled trials in each condition (as was done in the main analysis). This step was repeated 10,000 iterations to construct a distribution.**

**Our main interest was in the interaction of PK (PK/n-PK) by Memory (remembered/forgotten). To that end, we computed the interaction contrast ([PK:remembered-PK:forgotten]-[ n-PK:remembered-nPK:forgotten]) per participant and then averaged at the group level, per iteration. As can be seen in the figure below (left side), even with subsampling trials, 99.8% of the contrasts were lower than zero (note that since in the PK:remembered condition we found separation, i.e., a negative similarity values, we would expect the contrast to be negative). We have further tested the comparison between PK:remembered and n-PK:remembered, by computing the distribution of group average difference between these conditions (specifically, PK:remembered minus n-PK:remembered). We found that the group average differences in all 10,000 iterations were negative (right side graph on the figure below; negative values were expected due to the separation in the PK: remembered condition, as in the interaction contrast).**

Subsampling results. Left: Interaction contrast values (PK/nPK by remembered/forgotten). Right: PK vs. nPK differences for remembered trials. See text for more details. Dotted lines reflect the 95% confidence interval, The 0 line is marked in red.

**In addition, we examined whether the specificity to remembered trials was obtained in this analysis as well, in either the PK pairs or the n-PK pairs. Within each pair type, we computed the group average difference between remembered and forgotten pairs, in each of the 10,000 iterations. For PK pairs, 96.4% of the values were below zero, consistent with our main result that remembered pairs became less similar than forgotten. In the n-PK pair type, 98.6% of the values were above zero, consistent with our main result that for n-PK pairs, remembered pairs became more similar than forgotten.**

**Together, these results suggest that our findings are unlikely to be attributed to differences in the number of trials per condition. We thank the reviewer as we believe this additional analysis strengthened our results. We now report this analysis as detailed here in the Supplementary Information.**

7. It was not clear to me from reading the paper whether the change in representation was shown to be greater for PK vs. n-PK pairs (p. 8). This section says that there was change within the pairs, and that there was a difference between the paired items versus a shuffled baseline. However, this might be a difference observed only in the post-learning scan, rather than a difference in the amount of representational change. Please clarify and if it has not already been done, confirm that representational change is greater for paired items vs. the shuffled (within condition) control. (I believe the authors probably *have* done this, since that it how it is described later in the paper for a similar analysis.)

**We apologize for the unclarity – in this section we indeed refer to the change in representational similarity from before to after learning. We now include a sentence that specifically mention that. This paragraph now starts with the following: *“For robustness, and to further ensure that these effects in the left anterior hippocampus were specific to pairs that were learned together, rather than a general effect of observing faces with prior knowledge versus without prior knowledge, we compared remembered pairs to shuffled pairs (items from the same PK/n-PK pair-type that did not appear together at learning, see Methods). As above, we looked at the difference in similarity from before to after learning.”* (p. 8, the last sentence was added).**

8. The authors' approach of averaging beta-values across all pre-learning scans is not ideal as inflated similarity of the signal within-run will influence the pattern similarity values (and might bias the analyses, though I think this would depend on the specific orders). See Mumford, Davis & Poldrack (2014). These analyses should be changed to adopt this (what I would call) field standard approach, and all comparisons (correlations) restricted to betas derived from different scanning runs. It should at the very least be demonstrated and included in the paper whether the results stay the same when the analysis is performed in this way.

**We acknowledge the reviewer’s comment and we are aware of the approach of computing similarity across runs. This is a useful approach that has been successful in computing similarity for single-trial betas in some experimental designs. However, we believe that the concerns raised by Mumford et al. (2014) regarding autocorrelations between regressors tainting similarity values do not apply to our design, for two reasons: (1) We do not interpret raw similarity values, but rather, we compute a difference between similarity values obtained in the post-learning scans versus those obtained in the pre-learning scans, that had identical autocorrelations structure. (2) Mumford et al.’s concerns regard betas that are estimated based on a single trial, while we had multiple presentations per stimuli to estimate the betas corresponding to each stimulus. We elaborate on each of these points in turn below, after explaining our reasoning in computing stable activity patterns through averaging across 6 presentations of each stimulus.**

**As the reviewer mentions, in the current study we estimated each stimulus’ activity patterns based on 6 presentations per stimulus: we included 3 presentations per run and 2 runs per each pre-learning or post-learning phase. We then first average the beta values across runs, prior to computing the correlations. We reasoned that we should estimate the betas, and therefore the activity patterns, using as many stimulus presentations as possible, aiming for a stable estimate of the activity pattern, prior to computing the correlations. This is particularly important given that correlations are highly susceptible to outliers. Thus, we estimated as stable patterns as possible, that would in turn produce meaningful correlations.**

**Importantly, this reasoning was informed by the fact that we designed this study to look at similarity differences from before to after learning. As explained in the Methods (p. 36), the pre- and post-learning scans were identical. That ensured an identical autocorrelation structure in both parts of the study. Since we subtract the pre-learning similarity values from the post-learning values, any difference we observe cannot stem from the autocorrelation structure between regressors (Schapiro et al., 2012).**

**Additionally, Mumford et al.’s (2014) concerns arise when betas are estimated within runs based on a single trial. In this case, activity patterns might be influenced by autocorrelations between regressors (and mostly between neighboring stimuli). As mentioned above, we estimated our activity patterns based on 6 presentations per stimulus. We also emphasize that we first averaged the beta values obtained from the two runs in each phase, and then computed the correlation (i.e., per pair of stimuli, we had one pre-learning correlation value and one post-learning correlation value, rather than computing two correlation values for each phase, both within each run). Thus, our correlation values are not precisely within single runs, but rather based on betas that are obtained across different runs. Further, we randomized the trials order such that neighboring stimuli will change between stimuli presentations, and the correlation between regressors corresponding to the stimuli would be minimal. Moreover, Mumford et al. (2014) mentions that the concerns for single-trial betas are unlikely to apply to cases that trial order is randomized across participants. Even though we planned to estimate our betas based on 6 presentations and meant to look at similarity differences from pre- to post-learning, to equate the different conditions as much as possible, we counterbalanced across participants the allocation of stimuli and conditions (PK/n-PK, A/B-faces) to the pseudo-randomized trail orders we have created (see Methods). For all of these reasons, and mostly because we investigated differences from pre- to post-learning, we believe that the issues that regard single trial betas within runs do not apply to our design. We therefore chose to estimate our patterns based on as many stimulus presentations as possible.**

**Nevertheless, to address the reviewer’s comment, we have now additionally run the analysis computing correlations across runs, acknowledging that this might result in noisier estimates. We conducted this analysis for the hippocampus, since the asymmetry measure that we used in the left IFG analysis is computed across runs. In each pair of faces (A-face denotes the first face in the pair, B-face the second), and in each phase of the study (pre-learning, post-learning) we computed the correlation between the activity patterns of the A-face in the first run and the B-face in the second run, and the correlation between the activity patterns of the A-face in the second run and the B-face in the first run. We then average these values from the pre-learning runs to obtain a measure of the similarity pre-learning, and likewise for the post-learning runs. Then, we compute the difference between pre-learning and post-learning similarity, as was done in the main analysis. We further average similarity values by Memory (Remembered/Forgotten; remembered pairs included only high-confidence responses, as in the main text) and Pair Type (PK/n-PK).**

**Analyzing the data in this way, we obtained lower similarity for remembered PK pairs compared ton n-PK pairs (remembered: PK: *M* = -.02, *SD* = .10, n-PK: *M* = .13, *SD* = .20; *t_(17)_* = 3.11, *p* = .01), consistent with our main results. We also saw increased similarity for remembered n-PK pairs compared to forgotten pairs (forgotten n-PK: *M* = -.025, *SD* = .07; vs. remembered: *t_(17)_* = 3.12, *p* = .01). However, we no longer see the lower similarity for PK remembered pairs compared to forgotten pairs (forgotten PK: *M* = -.02, *SD* = .11; vs. remembered: *t_(17)_* = .02, *n.s.*). This result might mean that the activity patterns of the famous faces in the PK conditions are less stable across runs compared to the novel faces; therefore, when comparing across runs, we no longer see similarity differences between remembered and forgotten pairs. However, it might also mean that our result in the PK pairs were obtained based on patterns from one of the two runs in each pre/post-learning scans, but not from another run. We first wished to examine this possibility. To that end, we computed the similarity within each run, and then computed pre-learning to post-learning differences. Specifically, we correlated the activity patterns corresponding to pairs of faces in the first run (pre-learning), and subtracted these values from the similarity computed based on the first run (post-learning). Note that the order of the stimuli was identical across these runs, and similarity differences cannot be attributed to autocorrelations between regressors. Then, we did the same for the second runs in the pre-learning and post-learning. Again, the order of the stimuli was identical across these runs. These results are presented in the figure below. Obviously, since the patterns are computed based on half of the presentations, we expected worse estimates and noisier results. However, although expectedly non-significant, the pattern of the results is consistent with our main findings. In this analysis, the PK pairs consistently show qualitatively lower similarity for remembered versus forgotten pairs. Remembered PK pairs are also less similar compared to forgotten pairs in both runs. Thus, it does not seem like our PK results were obtained based on only one of the runs. In this analysis, the results in the n-PK pairs were less stable, as there is no difference between remembered and forgotten nPK pairs in the first run.**

**With some evidence that our main result, and especially in the PK pairs, does not stem from only one run, we went on to check the stability of representations across runs. We reasoned that activity patterns for famous faces might be less stable across runs, and that might contribute to not observing the memory effect within PK pairs in the analysis across runs above. To examine the stability of activity pattern across runs, we correlated the patterns of each face in the first run of the pre-learning task with the pattern of that same face in the second run of the pre-learning task, and we did the same for the post-learning task. Thus, we correlated each face with itself across runs (once within the pre-learning phase, and one within the post-learning phase). As these estimates were only based on half of the presentations, they were relatively noisy. As expected, both pre-learning and post-learning, remembered famous faces were qualitatively less similar to themselves than their parallel novel A-faces (pre-learning: PK: *M* = .009, *SD* = .09; n-PK: *M* = .019, *SD* = .16; post-learning: PK: *M* = -.029, *SD* = .11; n-PK: *M* = .007, *SD* = .16). Forgotten pairs did not exhibit a similar difference between PK and n-PK pairs consistently (pre-learning: PK: *M* = .02, *SD* = .06; n-PK: *M* = .013, *SD* = .05; post-learning: PK: *M* = .00, *SD* = .09; n-PK: *M* = .018, *SD* = .07). As can be seen in the standard deviations, these patterns indeed resulted in highly varied correlation values, and none of the differences reached significance (all *t*_(17)_*’*s < .76, *p*’s > .45).**

**Together, these analyses suggest that the patterns of famous faces might vary between runs, more so than novel faces. One speculation is that for famous faces, different memories are activated each time the participant is seeing these faces. These can be previous encounters with the famous face before the experiment or any information or memories that the participant has involving this famous person. If these memories differ between presentations of the famous face, this can reduce the similarity between patterns across runs. For novel faces, there are no such prior memories, and the only previous encounters are within the experiment, thus, if previous encounters are reactivated, that can contribute to stable representations of the novel faces across runs. We note, however, that all correlations computed were rather noisy, as can be seen in the standard deviations. To conclude, the variance we observed in computing the correlations across runs further strengthened our confidence that our approach of obtaining the activity patterns based on as many stimulus presentations as possible is essential to facilitate stable patterns and correlation values. Since the results in the additional analyses provided here were only qualitative, we currently chose not to include them in the manuscript. However, we are happy to include them in the Supplementary Information.**

Minor comments:

9. In the introduction, the authors make the case that prior knowledge might bias the hippocampus towards pattern separation (top of p. 5), but it is not clear to me why this would be the prediction. Couldn't it be just as easily predicted that the hippocampus pattern complete (e.g., to related associations and memories that exist for familiar but not novel faces)? Please clarify.

**We are in agreement that pattern completion can occur in the hippocampus when participants observe famous faces, potentially to mediate retrieval of pre-existing related associations. In our view, however, it is important to distinguish between the retrieval of previous associations and the encoding of novel associations. We now include this point in the Discussion, after discussing the associative inference studies: “*In light of the finding discussed above (Schlichting et al., 2015; Zeithamova & Preston, 2017), it is interesting to consider that prior knowledge might promote pattern completion to retrieve previous related memories, but then separation processes encode the novel association (Duncan, Sadanand, & Davachi, 2012; Hasselmo & Stern, 2014; Hasselmo, Wyble, & Wallenstein, 1996). Pattern completion refers to the retrieval of a previously encoded activity pattern from a partial cue (Knierim & Neunuebel, 2016; Marr, 1971; O’Reilly & McClelland, 1994; Treves & Rolls, 1994). Prior knowledge may facilitate hippocampal pattern completion to mediate the retrieval of pre-existing related association. Indeed, univariate activation is typically observed for famous faces compared to novel faces in the hippocampus (for reviews see Gobbini & Haxby, 2007; Natu & O’Toole, 2011). Nonetheless, this might not necessarily indicate that pattern completion occurs in the hippocampus. It is plausible that hippocampal activation drives pattern completion in other cortical regions where information might be stored (Bosch, Jehee, Fernandez, & Doeller, 2014; Danker, Tompary, & Davachi, 2017; Ritchey, Wing, LaBar, & Cabeza, 2013). It is further important to distinguish between pattern completion of previous associations and encoding of novel associations. Here, we have shown that separation might underlie the addition of a novel association to prior knowledge. One interesting possibility is that pattern completion may mediate the retrieval of previous associations and drive the hippocampal representations of the famous face, and then pattern separation may come into play and mediate the encoding of the novel association – to distinguish the novel association from previous memories. Otherwise, had the hippocampus remained in pattern completion ‘state’, interference with previous memories could have happened. Previous theoretical models and empirical work has shown that the switch from a retrieval ‘state’ that is potentially mediated by pattern completion, to an encoding ‘state’ that might require pattern separation, can be rapid in the hippocampus, and might be driven by the relative novelty elicited by the novel face in comparison to the famous face (Bein, Duncan & Davachi, 2019; Colgin, 2016; Duncan et al., 2012; Hasselmo, Bodelón, & Wyble, 2002; Hasselmo, Schnell, & Barkai, 1995)”* (pp. 20-21).**

**In the Introduction, we added a footnote referring to the Discussion: *“For a potential role of the hippocampus in pattern completion, namely, the recovery of a complete activation pattern from a partial cue, see Discussion.”* (p. 5).**

10. In the prior knowledge case, really only one thing needs to be learned (or maybe two; the new face + the link with the existing knowledge). To what extent is this driven by better, or more precise representation of the novel face that is part of the PK pairs? In other words, one might expect that since participants can spend more time encoding the novel face in these pairs, which could lead to a more specific representation of the items. Perhaps the authors have already performed an analysis that can rule this out.

**This is a valid point and one we have considered as well. However, we believe that the analyses performed in the manuscript render this possibility unlikely, due to the comparison to shuffled pairs. In all of the reported results, we compared the main result to shuffled pairs. As mentioned above, shuffled pairs consist of the same faces that were included in the main analysis, only we mixed the pairs such that the similarity was computed between faces that were not learned together during the associative learning task. Critically, faces were shuffled within pair type, such that shuffled pairs in the PK condition included only novel faces that appeared with famous faces during learning. If the results stemmed from novel faces that appeared in the PK pairs having more precise representations, then this should have influenced the shuffled pairs as well, as these same faces were used to compute shuffled-pairs similarity. Nevertheless, all the results were specific to pairs that appeared together during the study and did not occur for shuffled pairs. Thus, in our view, overall changes in representations of novel faces that appeared with PK pairs are unlikely to account for our results. In the section dedicated to alternative explanations, we now mention this possibility of a change in the representation of these faces: *“Another possibility is that the novel faces that were paired with famous faces in PK pairs carry with them some unique status due to becoming associated with famous faces, or that merely being paired with famous faces led to some overall change in the representation of these faces. However, again, any such status that is unrelated to associative learning would have been observed in the shuffled pairs as well”* (p. 15).**

11. Pattern similarity analyses were limited to correct (remembered) trials, whereas I believe PPI was performed across all trials. Please explain the reasoning behind this decision.

**This was done due to the number of stimuli we had in the experiment. Our main focus was the RSA analysis, and the number of pairs in each pair type (12 pairs in each) was determined to be sufficient for that analysis even when dividing them to remembered and forgotten, but within limitations of the duration of the experiment (Schapiro et al., 2012). In our pre-vs. post-learning RSA analysis, each face repeats multiple times, and the regressor-per-face in the GLM used to analyze the fMRI data includes multiple presentations of that face. Therefor the number of pairs we had is sufficient. However, the connectivity analysis is performed during the associative learning task, each pair is a single trial, and each regressor per pair type in the GLM includes all pairs within each pair type (note that we cannot collapse over repetitions at the level of the regressor, as that would include repetition of the same pair and thus samples will be dependent). Breaking these regressors further to remembered and forgotten pairs would leave us with too little trials for the analysis. Research in the past had examined the contribution of cortico-hippocampal connectivity to memory (Bein, Reggev, & Maril, 2014; Brod, Lindenberger, Wagner, & Shing, 2016; Liu, Grady, & Moscovitch, 2016; van Kesteren et al., 2010), and future research could continue to illuminate this important question.**

12. I assume the LIFG asymmetry analysis was performed only for remembered associations as well; please clarify.

**The asymmetry in the left IFG was calculated for all trials, as we did not find a difference in asymmetry from pre-learning to post-learning between remembered and forgotten pairs in the left IFG. We now report the asymmetry analysis by memory in the Supplementary Information: *“In the left inferior frontal gyrus, we found asymmetry in representational changes for PK pairs that were associated together during learning compared to shuffled pairs (Results, main text). We examined asymmetry separately for remembered and forgotten pairs. We found no evidence for asymmetry, as the asymmetry values for PK or n-PK pairs, either remembered or forgotten, did not significantly differ from zero or from each other (Remembered: PK: M = .002 , SD = .08, n-PK: M = -.007 , SD = .09; Forgotten: PK: M = .03 , SD = .07, n-PK: M = .01, SD = .07; all t’s_(17)_ < 1, p’s > .33, but Forgotten PK difference from zero: t_(17)_ = 1.92, p = .072). “***

**In the main text, we have now separated the analysis based on memory from the analysis based on all trials, and refer the reader to the supplementary for the asymmetry analysis: *“Regarding memory, in the left IFG the similarity differences from pre-learning to post-learning were not related to subsequent memory in the current study, as no main effect or interaction was obtained in the Prior Knowledge (PK/n-PK) by Memory (remembered/forgotten) ANOVA (all F’s < 2.46, p’s > .13). The asymmetry analysis for remembered and forgotten pairs is reported in the Supplementary Information.”* (p .13).**

**We hope these changes enhance the clarity of our results.**

13. Schlichting, Mumford & Preston (2015) found evidence for both separation and integration in the anterior hippocampus across different learning conditions, not integration overall as is implied on p. 16 of the discussion. The direction of representational change depended on the learning conditions, which might be useful to note in the present discussion (and related to the text in the immediately preceding paragraph).

**Thank you for this comment. We have modified the Discussion accordingly: *“The type of prior knowledge involved in new learning could be critical in shifting the hippocampus towards a separation mode versus an integration mode. Using an associative inference paradigm, a previous study showed that after an A-B pair was learned, learning of an overlapping A-C pair resulted in greater pattern similarity between the B and the C items in the anterior hippocampus (Schlichting et al., 2015, letters represent different items). Interestingly, this was only true if A-B pairs were repeated multiple times prior to learning the A-C pairs. If the learning of the A-C and A-B pairs was interleaved, the anterior hippocampus showed separation. Although they used merely visual associations, one can conceptualizing the A-B association as some prior knowledge to which a novel A-C association is added. As one could also think about the famous faces as have been learned over multiple repetitions prior to our study, our separation finding might seem to diverge from the previous results, showing similarity when A-B pairs were repeatedly learned prior to the A-C learning. This might point towards factors that bias hippocampal representations. For example, the A-B pairs form a single association learned over only a few repetitions, whereas the knowledge about the famous faces that we used in the current study is highly learned and involves a rich network of strong associations. Thus, one or a few weaker prior associations as in the case of the A-B pairs might not interfere with new learning and result in similarity (Schlichting et al., 2015), while multiple strong associations require interference-resolution and necessitate separation. However, it may also be the case that our presentation was more similar to the interleaved learning in Schlichting et al. (2015), since we reactivated the prior knowledge by presenting the famous face, along with presenting the novel face. Future research could examine how the type of knowledge as well as the learning protocol modulate hippocampal computations.”* (p. 19).**

14. At a couple of points in the paper the authors use the term "transitive inference" where I believe they mean "associative inference."

**Thank you. We have now replaced transitive inference with associative inference in all places in the manuscript.**

15. Were the faces in the n-PK condition on average matched in age with the PK pairs? I am wondering whether the same-age bias might mean the participants would have a better ability to represent the faces in the PK pairs if they are as whole closer in age to the participants than the n-PK stimuli.

**Yes. The A-faces in the n-PK pairs were age matched A-faces in the PK pairs, as mentioned in the Methods (p. 34). We had also selected our famous and novel faces to have a range of ages. Regarding B-faces, we randomly allocated these across pair type per participant. Moreover, as mentioned above, potential general differences in representing the faces themselves should have emerged in the shuffled-pairs as well. Nevertheless, we show that our results are specific to pairs that were learned together during the study. For these reasons, we do not believe that differences in age could account for our results.**

**Reviewer comments, second version:**

Reviewer #1 (Remarks to the Author):

The authors were thorough in responding to my concerns, and the paper is much improved. I have one remaining major comment. I asked the authors to report the pre and post similarity values in addition to the changes in similarity. They now do this in the supplement, adding strong caveats about its interpretability.

They report that in the pre-learning scans, similarity is higher for remembered pairs with prior knowledge (PK) than remembered pairs without prior knowledge (n-PK). This seems essential to the interpretation of the Figure 2 effects. It’s not that PK and n-PK pairs start off equal and then PK goes down and n-PK goes up. Rather, PK starts off higher and comes down, and n-PK starts off lower and comes up. The change is not driven entirely by the pre-learning values, but certainly this is part of the story. It seems essential to discuss this and provide a figure in the main text.

I am not following why these within session differences could be driven by regressor autocorrelation problems. If it is true that the regressors for these two different conditions have timecourses with different properties, that would seemingly lead to a range of problems. If not, then this is the kind of comparison that is routinely done in pattern similarity analyses.


Reviewer #2 (Remarks to the Author):

The changes made in response to the other comments make the manuscript significantly clearer. The authors are to be commended on their thoroughness in addressing the reviewers' comments. They've done a great job.

We have only one additional suggestion. Following major comment 1, we think it would be useful to include the analysis in the Supplementary and provide reasoning as to why this measure is noisier than others.


Reviewer #3 (Remarks to the Author):

The authors have done a fantastic (and thorough) job addressing my previous comments. The analyses they have done, many of which are included in the supplement, greatly strengthen the results. This is an excellent paper! My couple of remaining suggestions are:

- Additional motivation in the introduction for focusing on IFG, or even prefrontal cortex, specifically would be useful given the centrality of IFG in the results. The introduction is fairly general in its description of cortex, but I gather from the authors' response letter that IFG was meant to be the primary focus from the beginning. Right now, that is not coming through in the introduction.

- With respect to the title - I do appreciate that the authors have changed the terminology from "cortex" to "LIFG" in the abstract and section headings. I also do understand the desire for an elegant and punchy title. However, my recommendation would still be go with the slightly more verbose but accurate option. The effects are quite specific to LIFG it seems, which is interesting and, in my opinion, is getting lost in the general title about "cortex."

- It might be useful to highlight in the discussion that the asymmetry findings do not differ between remembered vs. forgotten pairs. How can this finding be reconciled with the notion that anchoring new information to prior knowledge promotes memory?

**Author rebuttal, second version:**

Reviewer #1 (Remarks to the Author):

The authors were thorough in responding to my concerns, and the paper is much improved. I have one remaining major comment. I asked the authors to report the pre and post similarity values in addition to the changes in similarity. They now do this in the supplement, adding strong caveats about its interpretability.

They report that in the pre-learning scans, similarity is higher for remembered pairs with prior knowledge (PK) than remembered pairs without prior knowledge (n-PK). This seems essential to the interpretation of the Figure 2 effects. It’s not that PK and n-PK pairs start off equal and then PK goes down and n-PK goes up. Rather, PK starts off higher and comes down, and n-PK starts off lower and comes up. The change is not driven entirely by the pre-learning values, but certainly this is part of the story. It seems essential to discuss this and provide a figure in the main text.

I am not following why these within session differences could be driven by regressor autocorrelation problems. If it is true that the regressors for these two different conditions have timecourses with different properties, that would seemingly lead to a range of problems. If not, then this is the kind of comparison that is routinely done in pattern similarity analyses.

**We acknowledge the reviewer’s suggestion, and we now address these findings in the main text. We also realize that our previous explanation might not have been detailed and clear enough, and we address this below. We do note, however, that while the current study is suitable to looking at similarity differences from pre-learning to post-learning, it was not designed to enable examining each phase separately. This is prevalent in previous studies that only report similarity differences, and do not interpret each phase separately**^1–3^**. Thus, albeit potentially interesting, we believe that these additional results from the pre-learning and post learning separately, obtained in a design that was not meant to specifically examine these data, and are only marginally significant, might be more suitable for the Supplementary Information. We hope that the current explanation better conveys our reasoning.**

**In the main text, we now report: *“While the current study is designed to examine differences from before to after learning***^2,3^***, we did not aim to specifically look at each of the pre-learning or post-learning phases separately, as these similarity values might be tainted by several factors like correlations between the regressors in the general linear model (GLM) used to analyze fMRI data***^4,5^***. Notably, since the scans before and after learning were identical, subtracting similarity values and looking at the differences, as was done in previous studies, solves these issues (see Methods, and refs.*** ^1–3^***). Nevertheless, to facilitate future research, we examined these values, and we note these results here, and in full in the Supplementary Note 5. As expected, we found that for remembered pairs, similarity values after learning were qualitatively lower in PK pairs compared to n-PK pairs. We additionally found that before learning, similarity values for remembered PK pairs were qualitatively higher than those of the remembered n-PK pairs, suggesting that prior knowledge might influence the preconditions that render associative learning successful and lead to subsequent memory of associations (see Supplementary Note 5). Future research, using a suitable design that enables a careful examination of the before and after learning values separately, potentially in a slow event-related design or other imaging techniques, could better elucidate these results.”* (pp. 7-8).**

**In Supplementary Note 5, we have modified the previous reporting of these results: *“In the main text, we present the difference between similarity before learning and similarity after learning. Here, we present the similarity values from the pre-learning and the post-learning scans separately, for completeness. We note that as previous studies examining pre- to post-learning differences in similarity***^1–3^***, the current study was not designed to look these similarity values separately. The similarity values before and after learning are influenced by several factors such as the sluggish nature of the BOLD signal, processing steps and correlations between the regressors in the fMRI GLM, that are dependent on the order of the stimuli as presented in the experiment***^4,5^***. Therefore, the raw similarity values are difficult to interpret at face value. Pattern similarity studies with various designs have different ways by which they address these issues, specific to each study and their aims. Previous studies that examined differences due to learning have typically addressed the aforementioned concerns by having the pre-learning and post-learning scans identical, subtracting the similarity values before learning from those values after learning, and reporting a difference in similarity^3–5^. This way, any influences from the BOLD signal, preprocessing, or correlations between GLM regressors are removed by the subtraction. The difference in similarity from before to after learning can therefore be interpreted with confidence. As detailed in the Methods, we followed the same approach in the current study. We further note that even though we did counterbalance the order of PK and n-PK faces across participants (see Methods), we still had no control over which pairs the participants later remembered or forgot.***

***Nevertheless, we acknowledge that it might be interesting to examine whether prior knowledge influence how neural patterns prior to associative learning might impact successful encoding, as measured by subsequent memory for the associated pair. To facilitate future research, we report here similarity values before and after learning separately, acknowledging that any findings, if emerges, should be confirmed in another design. As a reminder, when looking at differences from before to after learning, we found that for remembered pairs, the neural representations of faces in PK pairs became less similar to each other through learning, while the representations of faces in the n-PK pairs became more similar (Results, main text). When looking at similarity values in the post-learning scans, the similarity between remembered PK pairs was lower than in the n-PK pairs (PK: M = .02, SD = .07; n-PK: M = .09, SD = .12). We additionally found that in the pre-learning scans, the similarity was higher between remembered PK pairs compared to remembered n-PK pairs (PK: M = .08, SD = .10; n-PK: M = .10, SD = .11). Repeated-measures ANOVA, with the factors of Prior Knowledge (PK/n-PK) and Time Point (pre-learning/post-learning) revealed a significant interaction of Prior Knowledge and Time Point (F_(1,17)_ = 8.88, p = .008; simple-effects: pre-learning, PK vs. n-PK: t_(1,17)_ = 1.95, p = .07; post-learning, PK vs. n-PK: t_(1,17)_ = 1.90, p = .07; PK: pre- vs. post-learning: t_(1,17)_ = 3.01, p = .01; n-PK: pre- vs. post-learning: t_(1,17)_ = 2.08, p = .05). The same ANOVA for forgotten pairs revealed no main effect nor an interaction (all F_(1,17)_’s < 1.65, p’s > .21). Hippocampal neural patterns prior to learning have been shown to modulate subsequent learning in rodents and humans***^6–8^***. It is an interesting possibility that prior knowledge might modify the preconditions that determine whether future associative learning will be successful or not. However, even though our experiment was counterbalanced, for the reasons discussed above, it is currently unclear whether the neural patterns prior to learning reflect a true difference in neural similarity. Future research, using a methodology that enables a careful examination of the before and after learning values separately, potentially in a slow event-related design or other imaging techniques, could better elucidate these preliminary findings. Importantly, our results show that similarity differences from before to after learning cannot be attributed to differences before learning alone, as after learning, the similarity between remembered PK pairs was lower than n-PK pairs.”***

**We hope that the current report in the main text, together with our detailed explanation in the Supplementary Note 5, provides a fruitful reporting of the results, along with a clear explanation of the design and our inference.**

Reviewer #2 (Remarks to the Author):

The changes made in response to the other comments make the manuscript significantly clearer. The authors are to be commended on their thoroughness in addressing the reviewers' comments. They've done a great job.

We have only one additional suggestion. Following major comment 1, we think it would be useful to include the analysis in the Supplementary and provide reasoning as to why this measure is noisier than others.

**Thank you for this suggestion. We now report this analysis in the Supplementary Note 10, including an explanation of the analysis, the results, and reasons for why this analysis might be noisier: *“Representational changes for famous faces from pre-learning to post-learning in the hippocampus versus the left inferior frontal gyrus. In the main analysis in the hippocampus, we compared similarity between pairs of faces before and after learning. We found that the neural representations of famous and novel faces became more distinct, or more separated, from each other, through learning. An additional possible way to examine pattern separation in the hippocampus is to compute the similarity between the multivoxel activity pattern corresponding to a famous face prior to the associative learning after learning and compare that similarity value in the hippocampus to the same value in the left inferior frontal gyrus (left IFG). Note that this way of calculating similarity does not address separation between the famous face and its novel associated face, which was the focus of the current study. Nonetheless, it might be that the similarity of the same famous face from before to after learning would be lower in the hippocampus, due to pattern separation, compared to that in the left IFG.***

***To that end, we computed, for each famous face, the similarity between the multivoxel activity pattern before and after learning, in the left anterior hippocampus (where we found the main result) and in the left IFG. We took only remembered pairs, as these are the pairs that showed separation in the left anterior hippocampus (see Main text). We found that the similarity of famous faces from before to after learning was indeed lower in the left anterior hippocampus compared to the left IFG (Hippocampus: M = .003, SD = .09; left IFG: M = .017, SD = .05). However, as can be seen in the SDs, there was large variance in the data, and the qualitative difference did not reach statistical significance (t_(17)_ = .62, p = .52). It is possible that these values are noisier than our main analysis due to the time that has passed between the pre-learning and the post-learning scans (in which participants were doing the associative learning task). In the hippocampal results reported in the main text, similarity is computed between activity patterns that were obtained in the same phase, i.e., similarity is computed between faces in the pre-learning phase, and between faces in the post-learning, and then the difference in similarity is computed. Another possibility is that the separation we see in the hippocampus between a famous face and its associated novel face is a result of both the famous face and the novel face becoming distinct from one another, and thus examining only the famous face leads to statistically weaker results. This suggestion is consistent with the lack of asymmetry in hippocampal separation, as reported above (Supplementary Note 10). Thus, while the results of this analysis are generally consistent with our main findings, the specific manner by which prior knowledge promotes hippocampal separation is a topic for future investigation. “***

Reviewer #3 (Remarks to the Author):

The authors have done a fantastic (and thorough) job addressing my previous comments. The analyses they have done, many of which are included in the supplement, greatly strengthen the results. This is an excellent paper! My couple of remaining suggestions are:

- Additional motivation in the introduction for focusing on IFG, or even prefrontal cortex, specifically would be useful given the centrality of IFG in the results. The introduction is fairly general in its description of cortex, but I gather from the authors' response letter that IFG was meant to be the primary focus from the beginning. Right now, that is not coming through in the introduction.

**Thank you. We now specifically discuss the left IFG in the Introduction: *”Of a cohort of cortical regions that are involved in prior knowledge influences on new learning***^9^***, noteworthy are the left inferior frontal gyrus (left IFG), the Angular Gyrus (AG), and the medial prefrontal cortex (mPFC). We propose that the left IFG may be a good candidate to mediate the assimilation of novel information. Univariate activation in the left IFG has been consistently shown to promote memory of new information that is related to existing semantic knowledge***^10–12^***. The left IFG is also known to be involved in semantic processing more broadly***^13–16^***. The AG and mPFC have also been proposed to reinstate schematic knowledge and mediate prior knowledge influences on encoding, and thus might also be cortical sites for assimilation (for reviews, see refs.***^9,17^***).”* (pp. 4-5).**

- With respect to the title - I do appreciate that the authors have changed the terminology from "cortex" to "LIFG" in the abstract and section headings. I also do understand the desire for an elegant and punchy title. However, my recommendation would still be go with the slightly more verbose but accurate option. The effects are quite specific to LIFG it seems, which is interesting and, in my opinion, is getting lost in the general title about "cortex."

**The current title is: “*Prior knowledge promotes hippocampal separation but cortical assimilation in the left inferior frontal gyrus.”***

**This title complies with the editor’s request for less than 20 words and no punctuation.**

- It might be useful to highlight in the discussion that the asymmetry findings do not differ between remembered vs. forgotten pairs. How can this finding be reconciled with the notion that anchoring new information to prior knowledge promotes memory?

**We now address this in the Discussion:** ***“While in the current study we employed a memory test in which participants explicitly indicated the paired faces from the learning phase, other measures of memory might be more suitable for unveiling the benefits of neural assimilation. We note that using an explicit memory test, assimilation in the left IFG did not differ between subsequently remembered and forgotten famous-novel pairs (see Results, and Supplementary Note 7). This is a null result and thus should be interpreted with caution. However, an interesting possibility is that other, potentially more implicit measures, might better uncover the advantages of assimilation. For example, in associative priming paradigms, a presented item leads to a facilitation in the response to an ensuing item, as a result of these items being associated in memory***^18^***. If the neural representation of the novel face is assimilated into that of the famous face, it might be that upon encountering the famous face, the novel face is spontaneously reactivated, due to the similarity in their neural representation. That could potentially facilitate the response to that novel face. Indeed, we have recently shown that prior knowledge enhances associative priming, using a similar learning paradigm to the one employed here***^19^***. The explicit memory judgements that participants made in the current study likely relied on hippocampal representations and benefited from retrieval strategies that might overshadow the potential spontaneous reactivation that may have happened in the left IFG. This is consistent with the view that implicit and explicit forms of memory might rely on different mechanisms, and are potentially mediated by different neural systems***^20–22^***. The neural mechanisms by which prior knowledge might influence different forms of learning and memory are an exciting avenue for future research***^19^***.”* (pp. 21-22).**

*References*

1.	Kim, G., Norman, K. A. & Turk-Browne, N. B. Neural differentiation of incorrectly predicted memories. *J. Neurosci.* **37**, 2022–2031 (2017).

2.	Schapiro, A. C., Kustner, L. V. & Turk-Browne, N. B. Shaping of object representations in the human medial temporal lobe based on temporal regularities. *Curr. Biol.* **22**, 1622–1627 (2012).

3.	Schlichting, M. L., Mumford, J. A. & Preston, A. R. Learning-related representational changes reveal dissociable integration and separation signatures in the hippocampus and prefrontal cortex. *Nat. Commun.* **6**, 1–10 (2015).

4.	Mumford, J. A., Turner, B. O., Ashby, F. G. & Poldrack, R. A. Deconvolving BOLD activation in event-related designs for multivoxel pattern classification analyses. *Neuroimage* **59**, 2636–2643 (2012).

5.	Mumford, J. A., Davis, T. & Poldrack, R. A. The impact of study design on pattern estimation for single-trial multivariate pattern analysis. *Neuroimage* **103**, 130–138 (2014).

6.	Dragoi, G. & Tonegawa, S. Preplay of future place cell sequences by hippocampal cellular assemblies. *Nature* **469**, 397–401 (2011).

7.	Jafarpour, A., Piai, V., Lin, J. J. & Knight, R. T. Human hippocampal pre-activation predicts behavior. *Sci. Rep.* 1–9 (2017). doi:10.1038/s41598-017-06477-5

8.	Sadeh, T., Chen, J., Goshen-Gottstein, Y. & Moscovitch, M. Overlap between hippocampal pre-encoding and encoding patterns supports episodic memory. *Hippocampus* **29**, 836–847 (2019).

9.	Gilboa, A. & Marlatte, H. Neurobiology of Schemas and Schema-Mediated Memory. *Trends Cogn. Sci.* **21**, 618–631 (2017).

10.	Maril, A. *et al.* Event congruency and episodic encoding: A developmental fMRI study. *Neuropsychologia* **49**, 3036–3045 (2011).

11.	Reggev, N., Bein, O. & Maril, A. Distinct Neural Suppression and Encoding Effects for Conceptual Novelty and Familiarity. *J. Cogn. Neurosci.* **28**, 1455–1470 (2016).

12.	Staresina, B. P., Gray, J. C. & Davachi, L. Event congruency enhances episodic memory encoding through semantic elaboration and relational binding. *Cereb. Cortex* **19**, 1198–1207 (2009).

13.	Badre, D., Poldrack, R. A., Paré-Blagoev, E. J., Insler, R. Z. & Wagner, A. D. Dissociable controlled retrieval and generalized selection mechanisms in ventrolateral prefrontal cortex. *Neuron* **47**, 907–918 (2005).

14.	Badre, D. & Wagner, A. D. Left ventrolateral prefrontal cortex and the cognitive control of memory. *Neuropsychologia* **45**, 2883–2901 (2007).

15.	Binder, J. R. & Desai, R. H. The neurobiology of semantic memory. *Trends Cogn. Sci.* **15**, 527–536 (2011).

16.	Thompson-Schill, S. L., D’Esposito, M., Aguirre, G. K. & Farah, M. J. Role of left inferior prefrontal cortex in retrieval of semantic knowledge: A reevaluation. *Proc. Natl. Acad. Sci. U. S. A.* **94**, 14792–14797 (1997).

17.	van Kesteren, M. T. R., Ruiter, D. J., Fernández, G. & Henson, R. N. How schema and novelty augment memory formation. *Trends Neurosci.* **35**, 211–219 (2012).

18.	Schacter, D. L., Dobbins, I. G. & Schnyer, D. M. Specificity of priming: a cognitive neuroscience perspective. *Nat. Rev. Neurosci.* **5**, 853–62 (2004).

19.	Bein, O., Trzewik, M. & Maril, A. The role of prior knowledge in incremental associative learning : An empirical and computational approach. *J. Mem. Lang.* **107**, 1–24 (2019).

20.	Tulving, E. & Schacter, D. L. Priming and Human Memory Systems. *Science (80-. ).* **247**, 302–306 (1990).

21.	Squire, L. R. Memory systems of the brain: A brief history and current perspective. *Neurobiol. Learn. Mem.* **82**, 171–177 (2004).

22.	Reber, P. J. The neural basis of implicit learning and memory: A review of neuropsychological and neuroimaging research. *Neuropsychologia* **51**, 2026–2042 (2013).

**Reviewer comments, third version:**

**Author rebuttal, third version:**
